# The sphingosine kinase 2 inhibitors ABC294640 and K145 elevate (dihydro)sphingosine 1-phosphate levels in various cells

**DOI:** 10.1016/j.jlr.2024.100631

**Published:** 2024-08-23

**Authors:** Agata Prell, Dominik Wigger, Andrea Huwiler, Fabian Schumacher, Burkhard Kleuser

**Affiliations:** 1 Department of Pharmacology and Toxicology, Institute of Pharmacy, Freie Universität Berlin, Berlin, Germany; 2 Institute of Pharmacology, Inselspital, INO-F, University of Bern, Bern, Switzerland

**Keywords:** sphingolipids, phospholipids/metabolism, ceramides, lipidomics, phosphorylation, opaganib, COVID-19, off-target effects

## Abstract

Sphingosine kinases (SphKs), enzymes that produce the bioactive lipids dihydrosphingosine 1-phosphate (dhS1P) and sphingosine 1-phosphate (S1P), are associated with various diseases, including cancer and infections. For this reason, a number of SphK inhibitors have been developed. Although off-target effects have been described for selected agents, SphK inhibitors are mostly used in research without monitoring the effects on the sphingolipidome. We have now investigated the effects of seven commonly used SphK inhibitors (5c, ABC294640 (opaganib), N,N-dimethylsphingosine, K145, PF-543, SLM6031434, and SKI-II) on profiles of selected sphingolipids in Chang, HepG2, and human umbilical vein endothelial cells. While we observed the expected (dh)S1P reduction for N,N-dimethylsphingosine, PF-543, SKI-II, and SLM6031434, 5c showed hardly any effect. Remarkably, for K145 and ABC294640, both reported to be specific for SphK2, we observed dose-dependent strong increases in dhS1P and S1P across cell lines. Compensatory effects of SphK1 could be excluded, as this observation was also made in SphK1-deficient HK-2 cells. Furthermore, we observed effects on dihydroceramide desaturase activity for all inhibitors tested, as has been previously noted for ABC294640 and SKI-II. In additional mechanistic studies, we investigated the massive increase of dhS1P and S1P after short-term cell treatment with ABC294640 and K145 in more detail. We found that both compounds affect sphingolipid de novo synthesis, with 3-ketodihydrosphingosine reductase and dihydroceramide desaturase as their targets. Our study indicates that none of the seven SphK inhibitors tested was free of unexpected on-target and/or off-target effects. Therefore, it is important to monitor cellular sphingolipid profiles when SphK inhibitors are used in mechanistic studies.

Sphingolipids (SLs) are a class of both structural membrane components and bioactive mediators all containing a sphingoid base as building block. SLs are responsible for regulating many cellular processes such as cell survival and apoptosis, differentiation, migration, and immune reactions (1, 2, 3). Levels of SLs in the cellular compartments and circulation are strictly controlled by a complex system of enzymes. Consequently, alterations in SL metabolism play a crucial role in the pathogenesis of numerous diseases (4), e.g., cancer (5), neurological (6), metabolic (7), and cardiovascular (8) diseases, as well as viral (9) and bacterial (10) infections. The central molecules of the SL metabolism are ceramides (Cer), which originate from three key pathways: the de novo biosynthesis at the endoplasmic reticulum (ER), the hydrolysis of sphingomyelins (SMs) at the plasma membrane, and the salvage pathway comprising breakdown of complex SLs in lysosomal compartments and recycling of liberated sphingosine (Sph) (Fig. 1) (11). Alternatively, Sph can be phosphorylated by two isoforms of sphingosine kinases (SphK1 and SphK2) in an ATP-dependent reaction with the generation of sphingosine 1-phosphate (S1P) as a result (12). Although the SphK isoenzymes share some functions in the cell, they also have different roles that are determined by their subcellular localization and tissue distribution. Moreover, SphK2 has a larger substrate specificity and can also phosphorylate several artificial compounds that are not substrates for SphK1 (13, 14). However, the role of SphK2 is less understood and most of the identified S1P functions are regulated by SphK1, which is mainly located in the cytoplasm and translocated to the plasma membrane upon activation by phosphorylation (15). SphK2 is primarily located in the ER and mitochondria, where it regulates apoptosis, and in the nucleus, where it plays a role in epigenetic regulation (16, 17). SphK1 is highly expressed in the spleen, lung, and leukocytes, and SphK2 is highly expressed in the liver and kidney (14).

**Fig. 1:**
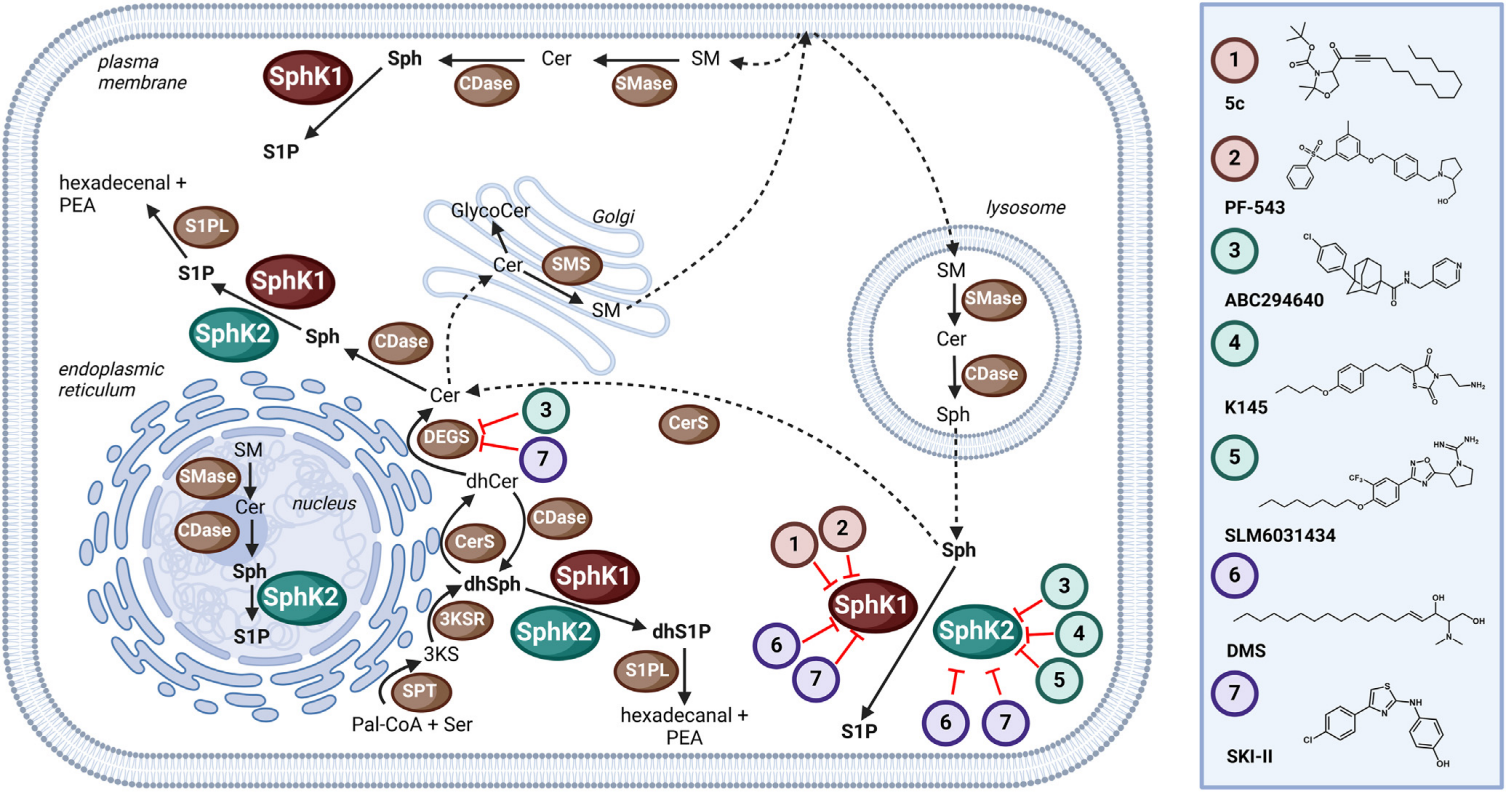
Overview of selected pathways of sphingolipid metabolism relevant to this study, as well as the specificity and previously reported off-target effects of the sphingosine kinase (SphK) inhibitors investigated (numbered 1–7, legend given on the right). 3KS, 3-ketodihydrosphingosine; 3KSR, 3-ketodihydrosphingosine reductase; CDase, ceramidase; Cer, ceramide; CerS, ceramide synthase; DEGS, dihydroceramide desaturase; dhCer, dihydroceramide; dhS1P, dihydrosphingosine 1-phosphate; dhSph, dihydrosphingosine; Pal-CoA, palmitoyl-coenzyme A; PEA, phosphoethanolamine; S1P, sphingosine 1-phosphate; S1PL, S1P lyase; Ser, l-serine; SM, sphingomyelin; SMase, sphingomyelinase; SMS, sphingomyelin synthase; Sph, sphingosine; SPT, serine palmitoyltransferase.

Over the past decades, numerous small molecule inhibitors have been designed to target one of the key enzymes in the SL metabolism. The majority of these target the biosynthesis of Cer (18), the central hub of the SL network. Three decades ago, the concept of the “sphingolipid rheostat” was developed, which describes the ability of S1P and Cer to differentially regulate cellular processes by modulating opposing signaling pathways (19). While Cer induces cell death, S1P is antiapoptotic (20, 21). Hence, the balance between both species is precisely regulated and determines cellular fate. SphKs are key regulators of the Cer-S1P rheostat as the only enzymes capable of phosphorylating Sph to produce S1P. SphKs have been implicated in various diseases including cancer, inflammatory disorders, cardiovascular disease, and infection (8, 22, 23, 24). In cancer, SphKs promote tumor cell proliferation, survival, and metastasis by generating S1P, which activates prosurvival signaling pathways and enhances angiogenesis (25). Both SphK1 and SphK2 have been shown to be upregulated in various human cancers (22). Similarly, some pathogens disrupt S1P metabolism by influencing the activity of the SphKs, mostly increasing it, since prolonged cell survival of infected cells is beneficial for the replication of microbial agents (26). Therefore, regulating the levels and activity of SphKs is a potential target in development of therapeutics (27). Accordingly, a variety of SphK inhibitors has been developed with a specificity against one or both isoforms (28). The existence of a crystal structure of SphK1 has favored the development of very effective SphK1 inhibitors. The situation is different for SphK2, for which no crystal structure is available to date. As a result, compounds with inhibitory effects on SphK2 have been developed whose efficacy is increasingly being questioned, which in turn has led to the continuous evolution of SphK inhibitor development and the current reports on increasingly potent and selective inhibitors (especially those of SphK2) (29). Five G-protein-coupled receptors (S1PR_1–5_) have been identified for the lipid messenger S1P (30). Dihydrosphingosine 1-phosphate (dhS1P) is also a ligand for S1P receptors, although the pharmacology and physiology of dhS1P is less well understood, presumably due to its much lower cellular concentrations compared to S1P (31). Consequently, numerous agonists and antagonists have been developed for one or more of these receptors. With fingolimod, ozanimod, siponimod, and ponesimod, the Food and Drug Administration and European Medicines Agency to date have approved the only four drugs modulating SL metabolism (32).

Interestingly, off-target effects were subsequently identified for various developmental substances or drug candidates in the framework of SL metabolism. For example, the first dihydroceramide desaturase (DEGS) inhibitor developed, GT11, was found to lose its specificity for this Cer-producing enzyme (IC_50_ of 23 nM) at higher concentrations, where it interfered with activities of serine palmitoyltransferase (SPT) and S1P lyase (33). The S1PR_2/4_ antagonist JTE-013 was recently found to inhibit DEGS1, as well as SphK1, and SphK2 at low micromolar concentrations (34). For the dual SphK1/2 inhibitors SKI-II and N,N-dimethylsphingosine (DMS), off-target effects within or outside the SL metabolic pathways have been demonstrated. While SKI-II inhibits DEGS (35), DMS is a potent protein kinase C inhibitor (36). Currently, the most advanced SphK inhibitor is ABC294640, also known as opaganib, which has been shown to exhibit antitumor, antiinflammatory, and antiviral activity (37). A recently concluded international phase 2/3 clinical trial of ABC294640 in COVID-19 hospitalized patients revealed that it could be safely administered to this patient group. Crucially, it led to a significant 62% reduction in mortality among a substantial subset of patients with moderately severe COVID-19 (https://doi.org/10.3390/microorganisms12091767). It is worth mentioning that for ABC294640, in addition to the described inhibitory effect on SphK2, off-target effects on SL metabolism were described, namely inhibition of DEGS and glucosylceramide synthase (37).

Given the central role of SphKs in the pathomechanism of some diseases, SphK inhibitors are widely used in basic research; however, most studies do not include sphingolipidome analysis or even S1P quantification, which may lead to misinterpretation of the data. Reports of important off-target effects such as inhibition of DEGS by ABC294640 and SKI-II, as well as divergent data and interpretations in the literature, have led us to believe that a more thorough characterization of SphK inhibitors is needed. Here, we describe the effects of seven commonly used SphK inhibitors (characteristics are summarized in Table 1) on profiles of selected sphingolipids in different cell lines and report novel off-target and unexpected on-target effects. For the two SphK2 inhibitors ABC294640 and K145, we performed additional mechanistic studies to explain the observed unexpected effects on the cellular SL profiles.

**Table 1:** Sphingosine kinase inhibitors included in this study and their properties

Inhibitor	CAS number	Kinase inhibited	K_i_ [μM]	Type of inhibition	Comments
DMS	119567-63-4	SphK1 SphK2	32 (38), 16 (39) 18 (38), 14 (39)	competitive (40)	targets other protein kinases (36, 41, 42).
SKI-II	312636-16-1	SphK1 SphK2	12 (38), 16 (39) 33 (38), 8 (39)	competitive (43), induces proteasomal degradation of SphK1 (44)	a noncompetitive inhibitor of DEGS (35), antitumor efficacy in animal models (45)
5c	120005-55-2	SphK1 SphK2	15 (38) 46 (38)	not known	therapeutic potential against cancer, endometriosis, has antisickling properties (46, 47, 48, 49)
PF-543	1706522-79-3	SphK1 SphK2	0.004 (50) 0.5 (50)	competitive (39), promotes proteasomal degradation of SphK1 (39, 51)	antiinflammatory, antifibrotic, and anticancer properties, potential therapeutic application in neurological, cardiovascular, fibrosis-related diseases, as well as cancer (52)
ABC294640 (Opaganib)	915385-81-8	SphK2	10 (53), 9.3 (39)	competitive (39), induces proteasomal degradation of SphK1 (54, 55)	inhibits DEGS (54) and glucosylceramide synthase (GCS) (37), partial antagonist to the estrogen receptor (54, 55), the only SphK inhibitor tested in humans, concluded phase 2/3 clinical trials for treatment of COVID-19 pneumonia and currently in multiple phase 1/2 studies for cancer (https://classic.clinicaltrials.gov/show/NCT04467840; https://classic.clinicaltrials.gov/show/NCT04207255; https://classic.clinicaltrials.gov/show/NCT03414489; https://classic.clinicaltrials.gov/show/NCT03377179; https://classic.clinicaltrials.gov/show/NCT02757326; https://classic.clinicaltrials.gov/show/NCT01488513)
K145	1449240-68-9	SphK2	6.4 (56)	competitive (56)	exhibits antitumor properties in vitro and in vivo (56)
SLM6031434	1897379-34-8	SphK2 SphK1	0.4 (57) 19 (57)	binds to the active site of SphK2 (58)	antifibrotic potential in mice (59)

Abbreviation: DMS, N,N-dimethylsphingosine.

## Materials and methods

### Chemicals

5c (Cat# S8326), DMS (Cat# SML0311), K145 hydrochloride (Cat# SML1003), PF-543 (Cat# 567741), SKI-II (Cat# S5696), and myriocin from Mycelia sterilia (Cat# M1177) were purchased from Sigma-Aldrich (Taufkirchen, Germany). SLM6031434 (Cat# 857381P) was from Avanti Polar Lipids (Alabaster), ABC294640 (Cat# 10587) and fumonisin B_1_ (FB_1_) (Cat# 62580) from Cayman Chemicals (Ann Arbor). Palmitic acid (16,16,16-d_3_) (Cat# CD2180P) was obtained from Cortecnet (Voisins-le-Bretonneux, France) and l-serine (2,3,3-d_3_) (Cat# D-1583) was from CDN Isotopes (Pointe-Claire, Canada). All SLs (unlabeled and deuterated) used as substrates for assays or reference standards for LC-MS/MS quantification were from Avanti Polar Lipids with the exception of C20 sphingomyelin (d18:1/20:0) and C22 sphingomyelin (d18:1/22:0) that were from Cayman Chemicals. LC-MS grade 1-butanol and chloroform were purchased from Sigma-Aldrich, while LC-MS grade acetonitrile and methanol were from VWR (Darmstadt, Germany). Recombinant human sphingosine kinases were purchased from Sigma-Aldrich (Cat# SRP0283, lot 4061090719 (rhSphK1) and Cat# SRP0285, lot 4061150713(rhSphK2)) and from R&D Systems (Minneapolis) (Cat# 5536-SK, lot SRC0722021 (rhSphK1) and Cat# 5298-SK, lot SIT0222121 (rhSphK2)). Ultrapure water was prepared using a water purification system from Sartorius Lab Instruments (Göttingen, Germany). All other chemicals and reagents were from Sigma-Aldrich unless otherwise noted. Stock solutions of SphK inhibitors were prepared in DMSO (Carl Roth, Karlsruhe, Germany).

### Cell culture

The human epithelial conjunctival (Chang) and the human hepatocyte carcinoma (HepG2) cell lines were cultured in RPMI-1640 medium with stable glutamine (BioWest, Düsseldorf, Germany) supplemented with 10% fetal calf serum (FCS) (Gibco, Thermo Fisher Scientific, Dreieich, Germany) and 1% penicillin-streptomycin. The human umbilical vein endothelial (HUVEC) cell line was cultured in EGM-2 medium (PromoCell, Heidelberg, Germany) containing 2% FCS. The proximal tubular (HK-2) cell line was cultured in DMEM:F12 medium (Gibco, Thermo Fisher Scientific) supplemented with 10% FCS and 1% penicillin-streptomycin. All control experiments were carried out under identical cultivation conditions compared to the treatment groups, including the amount of FCS added to the medium. The Chang, HepG2, and HK-2 cells were grown in 15 cm plates (Sarstedt, Nümbrecht, Germany). The HUVEC cells were grown in T75 flasks (Sarstedt). All cells were grown until 90% confluency at 37°C and 5% CO_2_ in a humidified atmosphere before being subcultivated. Chang cells were kindly provided by Dr Thomas Rudel (University of Würzburg, Germany). HepG2, HK-2, and HUVEC cells were obtained from ATCC (Manassas) and SphK1-deficient HK-2 cells were generated as described (60).

### Gene expression

Total RNA from cells was extracted with the RNeasy Mini Kit (Qiagen, Hilden, Germany). RNA concentration and purity were determined using the NanoPhotometer® N60 UV-Vis spectrophotometer (Implen, München, Germany). Only RNA with the ratio ∼2.0 of absorbance at 260/280 nm was used. The isolated mRNA was reverse-transcribed using the RevertAid Reverse Transcriptase (Thermo Fisher Scientific, Darmstadt, Germany, according to the manufacturer’s protocol). The quantitative reverse transcription-PCR was performed using the Maxima SYBR Green qPCR Mix (Thermo Fisher Scientific) on a LightCycler 480 II Real-Time PCR system (Roche, Mannheim, Germany). Quantification was done with the ΔΔ Ct method with *h-HMBS* as the reference gene. The stability of the reference gene expression was checked for every test substance. The oligonucleotide primers are listed in supplemental Table S1. All primers were tested with positive controls by performing melting profiles following quantitative reverse transcription-PCR and product sizes were checked by agarose gel electrophoresis. PCR conditions were as follows: initial denaturation at 95°C for 10 min, followed by 42 cycles of 15 s at 95°C, 15 s at annealing temperature (58°C) and 15 s at 72°C.

### Western blot analysis

Subsequently, 0.5 × 10^6^ of HUVEC cells or 2 × 10^6^ of Chang, HepG2, or HK-2 cells were seeded in 6 cm cell culture dishes and cultivated to reach 90% confluence. Afterward, cells were incubated with 50 μM ABC294640 or 10 μM K145 or vehicle for 5 h at 37°C. Cells were washed with ice-cold PBS and harvested by scraping in 250 μl ice-cold RIPA buffer (50 mM Tris–HCl (pH 7.5), 150 mM NaCl, 1% Triton X-100, 12 mM sodium deoxycholate, 0.1% SDS) supplemented with protease and phosphatase inhibitors. Cell suspensions were vortexed at highest level for 10 s and lysed in an ice-cold ultrasound bath for 10 min. Cell lysates were centrifuged at 9,400 *g* for 30 min at 4°C. Protein content was quantified by Bradford assay. Cell lysates were incubated (70°C, 10 min) in Laemmli buffer (Carl Roth), and 20 μg protein was used for SDS-PAGE. Gels were blotted onto nitrocellulose membranes. SphK1/2 expression and phosphorylation was then determined as previously described (61).

### Cell viability

Cytotoxicity of the SphK inhibitors investigated was determined using the 3-[4,5-dimethylthiazol-2-yl]-2,5 diphenyltetrazolium bromide (MTT) assay. Briefly, cells were seeded into 96-well plates at a density of 10,000 cells per well. Twenty-four hours after seeding, the cells were treated for 5 or 24 h with indicated concentrations of 5c, K145, SKI-II, DMS, ABC294640, SLM6031434, or PF-543. Positive controls were treated with 0.005% and 0.01% SDS, and to solvent controls volumes of DMSO equal to those of the incubations with the test substances (dissolved in DMSO) were added. Untreated controls were incubated for the same duration. After the incubation time, cells were washed with PBS and treated with 100 μl MTT solution per well (0.5 mg/ml in PBS) for 4 h at 37°C. Subsequently, supernatants were removed and 50 μl DMSO were added. The plates were shaken at 300 rpm for 10 min at room temperature. The optical density at 540 nm was measured using a microplate reader (Tecan, Männedorf, Switzerland). A cell viability <75% predicts cytotoxic effects.

### Cellular SphK inhibition assay

Briefly, 0.5 × 10^6^ of HUVEC cells or 2 × 10^6^ of Chang, HepG2, or HK-2 cells were seeded in 6 cm cell culture dishes and cultivated to reach 90% confluence. Afterward, cells were incubated with indicated concentrations of the SphK inhibitors 5c, K145, SKI-II, DMS, ABC294640, SLM6031434, or PF-543 for 5 h at 37°C. For the coincubation experiments, cells were treated with K145 or ABC294640 in combination with PF-543 at indicated concentrations for 5 h at 37°C. Solvent controls were treated with volumes of DMSO equal to those of the applied inhibitor working solutions. After the incubation, media were removed. Cells were washed with ice-cold PBS, harvested by scraping in 0.5 ml ice-cold methanol and subjected to lipid extraction for SL quantification by LC-MS/MS.

### Cell-free SphK1 and 2 inhibition assay

The inhibitory effect of the SphK inhibitors was tested on recombinant human (rh) enzymes from two manufacturers (Sigma-Aldrich and R&D Systems). Briefly, the assay mixture with a total volume of 500 μl contained 0.05 μg rhSphK1 or 0.0625 μg rhSphK2, 1 μM Sph, and 4 mM ATP as substrates of the reaction, as well as 5 mM β-glycerophosphate, 1 mM calcium chloride (CaCl_2_), cOmplete™ protease inhibitor (2.7-fold), 5 mM sodium orthovanadate (Na_3_VO_4_), 160 μM coenzyme A (CoA) (Cayman Chemicals), 0.8 mM dithiothreitol (DTT), 40 mM 4-(2-hydroxyethyl)-1-piperazineethanesulfonic acid (Hepes), 2 mM magnesium chloride (MgCl_2_), and 121 mM sodium chloride (NaCl) (Carl Roth). After addition of SphK inhibitors at indicated concentrations, the mixtures were incubated for 1 h at 37°C under gentle shaking (120 rpm). Corresponding volumes of DMSO served as vehicle controls. Reactions were stopped by addition of methanolic KOH and SLs were extracted with 1-butanol (containing d_7_-sphingosine (d_7_-Sph) and d_7_-sphingosine 1-phosphate (d_7_-S1P) as internal standards). Vacuum-dried lipid extracts were resuspended in 100 μl acetonitrile/methanol/water (47.5:47.5:5 (v:v:v), 0.1% formic acid) and subjected to LC-MS/MS analysis.

### Cellular DEGS inhibition assay

In addition, 0.5 × 10^6^ of HUVEC cells or 2 × 10^6^ of Chang or HepG2 cells were seeded in 6 cm cell culture dishes and cultivated. Upon reaching a 90% confluence, cells were preincubated with the SphK inhibitors 5c, K145, SKI-II, DMS, ABC294640, SLM6031434, or PF-543 at indicated concentrations for 1 h at 37°C. For the remaining 4 h of incubation, 1 μM of the stable isotope-labeled DEGS substrate d_7_-C13:0 dihydroceramide (dhCer) was added. Solvent controls were treated with volumes of DMSO equal to those of the applied inhibitor working solutions. After the incubation, media were removed and cells were washed with ice-cold PBS, harvested by scraping in 0.5 ml ice-cold methanol and subjected to lipid extraction for LC-MS/MS quantification.

### Cellular sphingolipid de novo synthesis assay

Briefly, 2 × 10^6^ of Chang cells were seeded in 6-cm cell culture dishes and cultivated to reach 90% confluence. Afterward, cells were preincubated with indicated concentrations of the SphK2 inhibitors K145 or ABC294640 for 1 h at 37°C. For the remaining 4 h of the assay, 10 μM of stable isotope-labeled d_3_-palmitate was added. Solvent controls were treated with volumes of DMSO equal to those of the applied inhibitor working solutions. After the incubation, media were removed. Cells were washed with ice-cold PBS, harvested by scraping in 0.5 ml ice-cold methanol and subjected to lipid extraction for SL quantification by LC-MS/MS.

### Cell-free sphingolipid de novo synthesis assay

The microsomal SL de novo assay was performed according to our previously published protocol with few modifications (62). Instead of rat liver microsomes as described, we used 50 μg of human liver microsomes (Cat# M0567, Sigma-Aldrich). Briefly, the assay mixture contained microsomes as source of ER-bound SL metabolizing enzymes (SPT, 3-ketodihydrosphingosine reductase [3KSR], ceramide synthase [CerS] and DEGS), d_3_-palmitate and d_3_-l-serine as deuterated substrates of SL de novo synthesis as well as all required cofactors, reducing equivalents, and buffers. After addition of 50 μM ABC294640, 10 μM K145, or 5 μM FB_1_, mixtures were incubated for 1 h at 37°C under gentle shaking (120 rpm). In a modified version of this assay, we replaced deuterated SL precursors with 3-ketodihydrosphingosine (3KS) and blocked SPT catalysis by adding myriocin. The assay was then performed with 50 μM ABC294640 or 10 μM K145 in the presence or absence of 5 μM FB_1_. Corresponding volumes of DMSO served as vehicle controls. Reactions were stopped by addition of methanolic KOH and SLs were extracted with 1-butanol (containing 17:0 ceramide (C17:0 Cer) and C17 dihydrosphingosine (C17 dhSph) as internal standards). Vacuum-dried lipid extracts were resuspended in 100 μl acetonitrile/methanol/water (47.5:47.5:5 (v:v:v), 0.1% formic acid) and deuterium label-bearing, de novo-formed SL species or dhSph were analyzed by LC-MS/MS as described below.

### Determination of intracellular DEGS activity by LC-MS/MS

Suspensions of cells incubated with the stable isotope-labeled DEGS substrate d_7_-C13:0 dhCer were subjected to lipid extraction as described in the next section for determination of the cellular sphingolipidome. The extraction solvent, however, contained only C17:0 Cer and d_31_-C16:0 SM as internal standards. For the analysis, a 1290 Infinity II HPLC coupled with a 6465B (Ultivo) triple-quadrupole mass spectrometer (both Agilent Technologies, Waldbronn, Germany) was used. Chromatographic conditions including eluents used and gradient program (supplemental Table S2), separation column, temperature of column compartment and injection volume were as described in the following paragraph. Settings of the electrospray ion source operated in positive mode (ESI+) were as follows: sheath gas temperature, 375°C; sheath gas flow, 12 L/min of nitrogen; nebulizer pressure, 30 psi; drying gas temperature, 200°C; drying gas flow, 13 L/min of nitrogen; capillary voltage, 4,000 V; and nozzle voltage, 1,500 V. A total of 14 multiple reaction monitoring transitions (dwell time: 75 ms each) were applied to monitor cellular DEGS activity (supplemental Table S3). To this end, peak areas of the substrate d_7_-C13:0 dhCer as well as the product d_7_-C13:0 Cer were normalized to the internal standard C17:0 Cer and quantified via C16:0 Cer external calibration using MassHunter Quantitative Analysis software (version 10.1, Agilent Technologies; https://www.agilent.com/en/product/software-informatics/mass-spectrometry-software/data-analysis/quantitative-analysis). C18:0 SM (quantified via d_31_-C16:0 SM) as an abundant component of plasma membranes was comeasured to monitor the use of comparable cell material for the assay. Intracellular DEGS activity was determined as conversion rate (%) = c (d_7_-C13:0 Cer)/(c (d_7_-C13:0 dhCer) + c (d_7_-C13:0 Cer)) with c being the concentration (in nM) in the lipid extract.

### Extraction and quantification of sphingolipids by LC-MS/MS

Cell suspensions were subjected to lipid extraction using 1.5 ml methanol/chloroform (2:1, v:v) containing d_7_-dihydrosphingosine (d_7_-dhSph), d_7_-sphingosine (d_7_-Sph), d_7_-sphingosine 1-phosphate (d_7_-S1P), 17:0 ceramide (C17:0 Cer) and d_31_-16:0 sphingomyelin (d_31_-C16:0 SM) as internal standards. Extraction was facilitated by incubation at 48°C with gentle shaking (120 rpm) overnight. To reduce interference from glycerolipids, samples were saponified with 150 μl 1 M methanolic KOH for 2 h at 37°C with gentle shaking (120 rpm) followed by neutralization with 12 μl glacial acetic acid. After centrifugation at 2,200 *g* for 10 min at 4°C, organic supernatants were evaporated to dryness using a Savant SpeedVac concentrator (Thermo Fisher Scientific). Dried residues were reconstituted in 200 μl acetonitrile/methanol/water (47.5:47.5:5 (v:v:v), 0.1% formic acid) and subjected to LC-MS/MS quantification applying the multiple reaction monitoring approach. Chromatographic separation was achieved on a 1,290 Infinity II HPLC (Agilent Technologies) equipped with a Poroshell 120 EC-C8 column (3.0 × 150 mm, 2.7 μm; Agilent Technologies) guarded by a precolumn (3.0 × 5 mm, 2.7 μm) of identical material. The column compartment was maintained at 30°C and the injection volume was 10 μl. A mobile phase system consisting of water (solvent A) and acetonitrile/methanol (1:1, v:v; solvent B), both acidified with 0.1% formic acid, was used for gradient elution at an initial composition of 40:60 (A:B, v:v) and a flow rate of 0.5 ml/min (supplemental Table S2). MS/MS analyses were carried out using a 6495C triple-quadrupole mass spectrometer (Agilent Technologies) operating in the ESI+ mode. The following ion source parameters were set as follows: sheath gas temperature, 375°C; sheath gas flow, 12 L/min of nitrogen; nebulizer pressure, 20 psi; drying gas temperature, 240°C; drying gas flow, 20 L/min of nitrogen; capillary voltage, 4,000 V; nozzle voltage, 2,000 V; iFunnel high pressure RF voltage, 90 V; and iFunnel low pressure RF voltage, 60 V. For the determination of canonical SLs, 67 mass transitions were recorded; the dwell time was 13 ms each. After incubation of cells with d_3_-palmitate and additional determination of de novo-formed deuterated lipids, a total of 130 mass transitions was recorded with a dwell time of 5 ms each (supplemental Table S4). Peak areas of Cer, dhCer, SM, and dihydrosphingomyelin (dhSM) subspecies were normalized to those of their internal standards (C17:0 Cer for Cer and dhCer subspecies; d_31_-C16:0 SM for SM and dhSM subspecies) followed by external calibration in the range of 1 fmol–5 pmol (dhCer) or 50 pmol (Cer, SM, dhSM) on column. DhSph, Sph, and S1P were directly quantified via their deuterated internal standards d_7_-dhSph (0.125 pmol on column), d_7_-Sph (0.25 pmol on column) and d_7_-S1P (0.125 pmol on column). DhS1P was quantified via d_7_-S1P. Whenever dhCer and Cer levels are mentioned and it is not explicitly stated otherwise, this refers to the sum of the subspecies (C16:0, C18:0, C20:0, C22:0, C24:0, and C24:1). In extracts of the microsomal de novo synthesis assay, the following analytes were quantified, d_5_-3KS, d_5_-dhSph, and d_8_-C16:0 dhCer. To this end, peak areas were normalized to those of their internal standards (C17 dhSph for d_5_-3KS, d_5_-dhSph and C17:0 Cer for d_8_-C16:0 dhCer) and quantified via 3KS, dhSph, and C16:0 dhCer external calibration curves (range: 1–1,000 nM), respectively. Detailed LC-MS/MS parameters are given in supplemental Table S5. Data evaluation was performed with MassHunter Quantitative Analysis software (version 10.1, Agilent Technologies).

## Results

### Unexpected effects of SphK inhibitors on cellular S1P and dhS1P levels

**Fig. 2:**
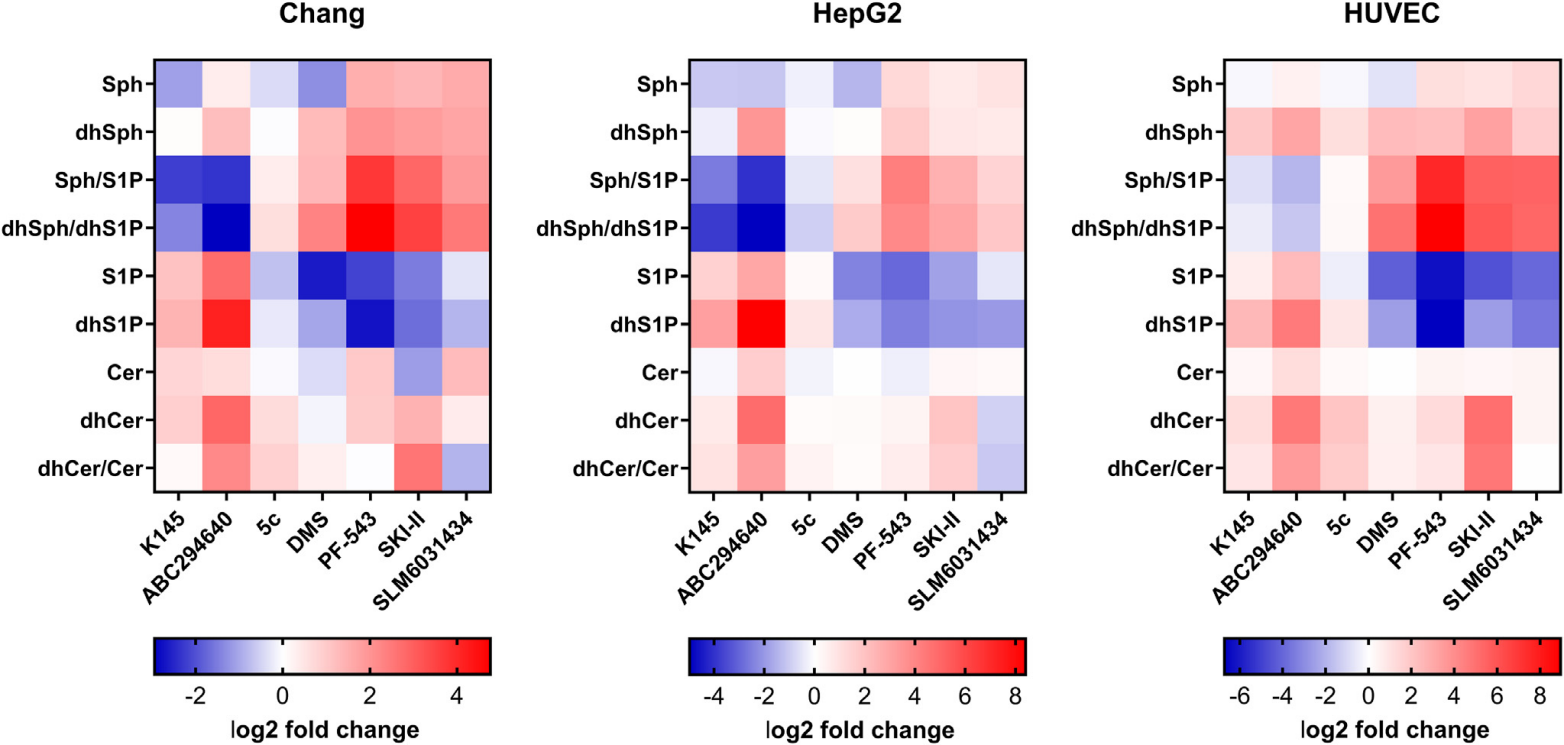
Relative changes (log2 fold) of selected sphingolipid species and ratios, determined by LC-MS/MS, after treatment of Chang, HepG2, and HUVEC cells for 5 h with sphingosine kinase inhibitors compared to corresponding solvent controls. The individual inhibitor concentrations were either 10 μM for all inhibitors (but 50 μM for ABC294640) for Chang and HepG2 cells, or 5 μM (SLM6031434, K145, N,N-dimethylsphingosine), 10 μM (5c, PF-543, SKI-II), or 25 μM (ABC294640) for HUVEC cells. The heatmaps are based on the detailed lipidomics data presented in supplemental Figs. S4–S8. Cer, ceramide; dhCer, dihydroceramide; dhS1P, dihydrosphingosine 1-phosphate; dhSph, dihydrosphingosine; HUVEC, human umbilical vein endothelial; S1P, sphingosine 1-phosphate; Sph, sphingosine.

In order to perform a comprehensive inhibition study, we selected three different cell lines: human conjunctival cells (Chang), human liver carcinoma cells (HepG2), and human umbilical vein endothelial cells (HUVEC). First of all, we checked whether all three cell lines express both SphK isoforms using qPCR. As can be seen from supplemental Fig. S1, *SPHK1* and *SPHK2* mRNA could be detected in any case. However, *SPHK1/SPHK2* ratios differed between cell types. Next, we performed sphingolipidome analyses with the three cell lines using LC-MS/MS (supplemental Fig. S2). The data show that the long-chain bases (LCBs), dhSph and Sph as well as their phosphorylated metabolites dhS1P and S1P, were clearly detectable in the cellular test systems. Remarkably, there were significant differences in basal LCB levels between the cell lines. We proceeded with the inhibition study, for which we used seven established SphK inhibitors, two of which are selective for SphK1 (5c and PF-543), three are selective for SphK2 (ABC294640, K145, and SLM6031434) and two others are dual SphK1/2 inhibitors (SKI-II and DMS) (Fig. 1). In a dose-response experiment, the cells were incubated with the inhibitors for 5 h. All inhibitor concentrations used were nontoxic to the cells as verified by MTT cell viability assays (supplemental Fig. S3). The heatmaps presented in Fig. 2, show the observed effects of SphK inhibitors on selected cellular SL species at the highest individual inhibitor concentration used. The detailed dose-dependent effects of the test compounds on the cellular (dh)Sph-SphK-(dh)S1P axis can be found in supplemental Figs. S4–S6. As expected, we observed a decrease in S1P and dhS1P for DMS, PF-543, SKI-II, and SLM6031434 compared to the solvent control across cell lines. Constantly, PF-543 showed the strongest inhibitory effect. With the exception of DMS, the decline in S1P and dhS1P was accompanied by rising concentrations of Sph and dhSph, respectively, resulting in a marked increase in the ratios of Sph/S1P and dhSph/dhS1P for the abovementioned four inhibitors in the cell lines investigated. The SphK1-specific inhibitor 5c showed the weakest effects of all tested compounds across cell lines. A weak but statistically significant decrease in S1P was only observed in HUVEC cells. The most remarkable effects of this inhibition study resulted for the two SphK2 inhibitors K145 and ABC294640. Contrary to expectations, incubations of Chang, HepG2, and HUVEC cells with ABC294640 or K145 resulted in highly significant increases in cellular S1P (Fig. 2). Compared to solvent controls S1P increased 2.2-fold (Chang), 2.9-fold (HepG2), and 1.5-fold (HUVEC) for K145 as well as 6.7-fold (Chang), 7.3-fold (HepG2), and 5.2-fold (HUVEC) for ABC294640. These unexpected effects were even more pronounced when considering dhS1P that rose 2.6-fold (Chang), 10.0-fold (HepG2), and 5.5-fold (HUVEC) after incubation with K145, and even 17.8-fold (Chang), 338-fold (HepG2), and 24.3-fold (HUVEC) in the presence of ABC294640. While K145 and ABC294640 increased S1P in all cell lines, Sph levels behaved more heterogeneously. Interestingly, the striking increase in dhS1P was associated with significantly elevated dhSph levels across cell lines after treatment with ABC294640. For K145 treatment, this only applied to HUVEC cells. We summarize our observations regarding the influence of the seven SphK inhibitors tested on the cellular (dh)Sph/SphK/(dh)S1P axis as follows: *1)* PF-543 (specific for SphK1), SLM6031434 (specific for SphK2), DMS, and SKI-II (both dual SphK1/2 inhibitors) caused (dh)S1P reduction in all cells, as expected, whereas 5c (specific for SphK1) was virtually ineffective in this regard. *2)* ABC294640 and K145 (both specific for SphK2) caused the opposite and dramatically increased S1P and dhS1P across cell lines. *3)* Especially after treatment with ABC294640, significant increases in dhSph were observed.

### SphK inhibitors affect cellular DEGS activity

**Fig. 3:**
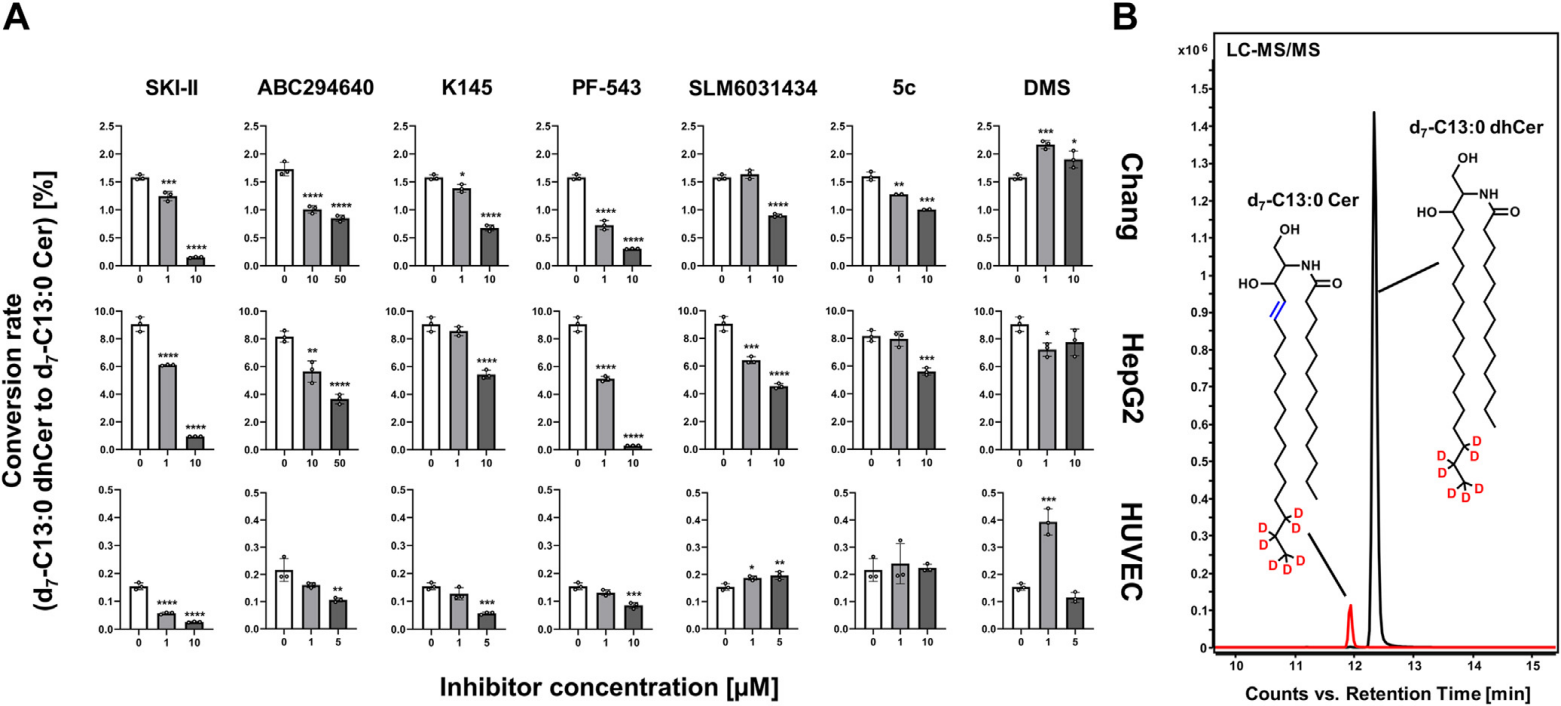
Effects of sphingosine kinase inhibitors on dihydroceramide desaturase (DEGS) activity in Chang, HepG2, and HUVEC cells. A: Cells were incubated with indicated concentrations of inhibitors together with 1 μM deuterated DEGS substrate for 5 h. Both, d_7_-C13:0 dihydroceramide (dhCer, substrate) and d_7_-C13:0 ceramide (Cer, product) were quantified by LC-MS/MS and the dhCer-to-Cer conversion rate was calculated. Shown is the mean ± SD of three independent experiments (with the exception of 1 and 10 μM 5c in Chang cells, n = 2). Statistically significant differences to the solvent control (white bars) were determined using one-way ANOVA with Dunnett's multiple comparison test. ∗*P* < 0.05, ∗∗*P* < 0.01, ∗∗∗*P* < 0.001, ∗∗∗∗*P* < 0.0001. B: LC-MS/MS chromatogram for the detection of both substrate and product of the monitored DEGS reaction. Chemical structures of the analytes are given as insets. DEGS, dihydroceramide desaturase; HUVEC, human umbilical vein endothelial.

The SphK2 inhibitor ABC294640 (63) and the dual SphK1/2 inhibitor SKI-II (35) have already been described to inhibit DEGS activity. The cell lines central to this study all expressed *DEGS1* mRNA (supplemental Fig. S1). Moreover, we included dhCer and Cer in our sphingolipidomic analysis (supplemental Fig. S2), which allowed us to investigate effects of SphK inhibitors on the cellular dhCer-Cer axis. Following inhibitor treatment, we were able to confirm the reported suppressive effects of ABC294640 and SKI-II on DEGS activity with increased dhCer levels and dhCer/Cer ratios across cell lines (Fig. 2 and supplemental Fig. S7). Strikingly, we also observed effects, albeit weaker, on the dhCer-Cer axis with other SphK inhibitors tested, thus uncovering putative hitherto unknown inhibitory effects on DEGS. As such, 5c, almost ineffective in lowering S1P levels in our studies, significantly increased the dhCer/Cer ratio in all cell lines, with the strongest manifestation in HUVEC cells. K145 not only caused similar, even though less pronounced, unexpected effects on (dh)S1P levels as ABC294640, but likewise increased dhCer/Cer ratios, at least in HepG2 and HUVEC cells. Of further note, SLM6031434 reduced the dhCer/Cer ratio by up to approximately 50% in a dose-dependent manner in Chang and HepG2 cells. DMS and PF-543 also tended to increase dhCer/Cer, but no clear picture emerged across the concentration range and cell line. For ABC294640 and K145, supplemental Fig. S8 exemplifies the influence of treatment on SL subspecies (*N*-acyl chain length from C16:0 to C24:1) in Chang cells. We then attempted to corroborate these findings by more accurately determining cellular DEGS activity using an alternative targeted approach. To this end, we preincubated cells with the SphK inhibitors of interest for 1 h before incubating the stable isotope-labeled DEGS substrate d_7_-C13:0 dhCer together with the inhibitors for additional 4 h. We then quantified both the substrate and the product that can be formed exclusively by DEGS activity, d_7_-C13:0 Cer by LC-MS/MS (Fig. 3B). Again, we were able to confirm the described DEGS inhibition by ABC294640 and SKI-II with a dose-dependent reduction of the dhCer-to-Cer conversion rate across cell lines (Fig. 3A). Even more interestingly, effects on DEGS activity were also observed for all five other SphK inhibitors. It was most evident, as observed in all cells, for K145 and PF-543. Both compounds significantly inhibited DEGS activity in a dose-dependent manner. SLM6031434 inhibited DEGS catalysis starting at 1 μM in HepG2 and 10 μM in Chang cells. In HUVEC cells, however, a slight but significant increase in dhCer-to-Cer conversion was observed starting at 1 μM. Subsequently, 5c significantly reduced DEGS activity in Chang and HepG2, starting at 1 μM and 10 μM respectively, but not in HUVEC cells. DMS was the only inhibitor to show a biphasic dose-response relationship in the DEGS activity assay. The lower concentration (1 μM) increased DEGS activity in Chang and HUVEC cells, while the next higher concentration (10 μM in Chang, 5 μM in HUVEC) weakened or even abolished this effect. For HepG2 cells, the assay revealed negligible effects of DMS on DEGS activity. It can be summarized that *1)* the published DEGS inhibition by ABC294640 and SKI-II was confirmed across assays. *2)* Varying degrees of dose-dependent, so far undescribed, inhibition of DEGS activity were demonstrated for all other inhibitors (except DMS). *3)* The data from the untargeted inhibition analysis (Fig. 2) and the specific cellular DEGS activity assay (Fig. 3) were only partially concordant, indicating a more complex metabolic interplay (than just DEGS catalysis) affecting the dhCer-Cer axis.

### ABC294640 and K145 attenuate the (dh)S1P-reducing effects of PF-543

**Fig. 4:**
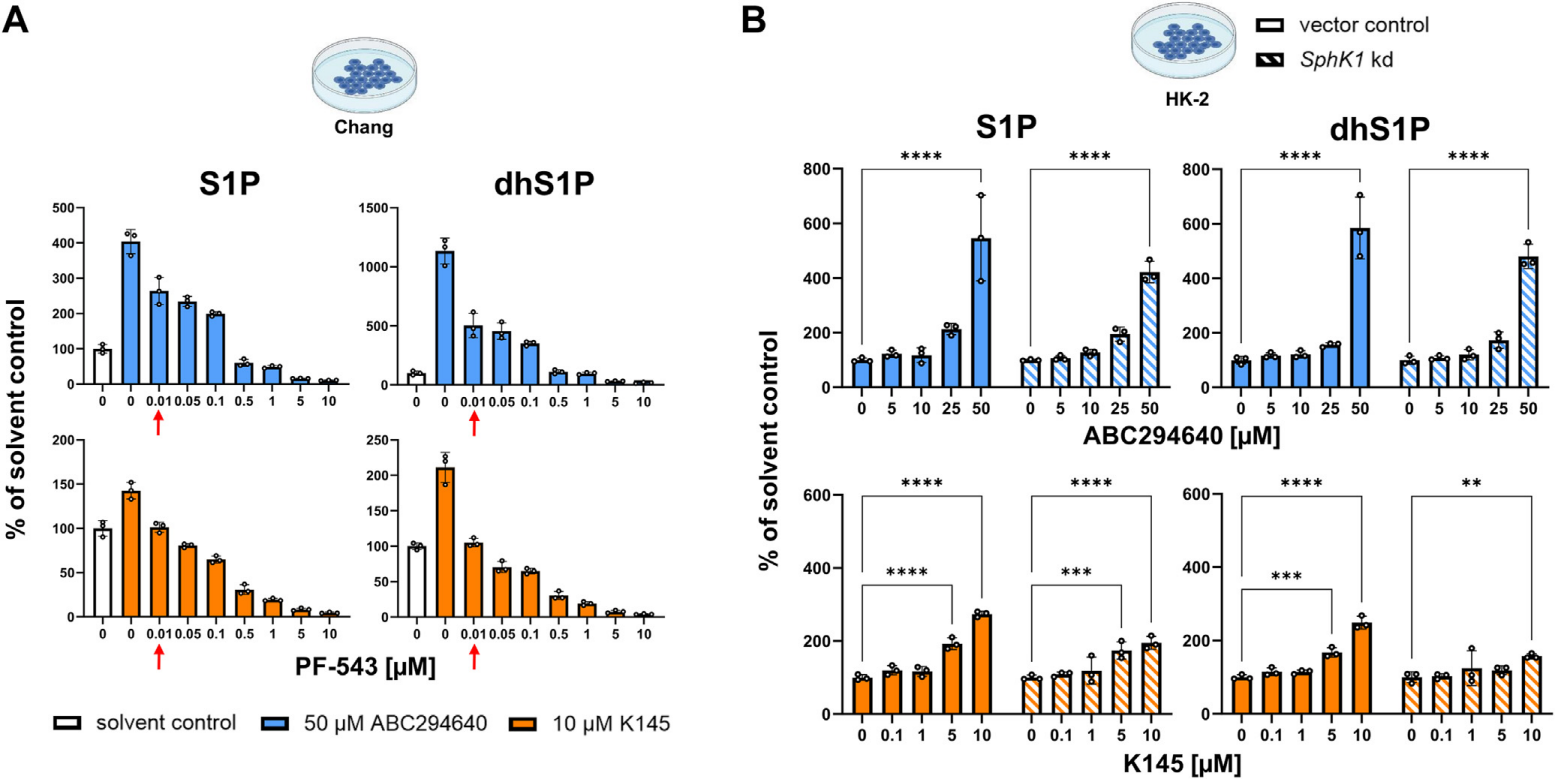
Contribution of sphingosine kinase (SphK) 1 to increased cellular sphingosine 1-phosphate (S1P), and dihydrosphingosine 1-phosphate (dhS1P) levels after treatment with ABC294640 or K145. A: Chang cells incubated with 50 μM ABC294640 or 10 μM K145 were costimulated with indicated concentrations of the SphK1-specific inhibitor PF-543 for 5 h. S1P and dhS1P were analyzed by LC-MS/MS, and the levels are given relative to the solvent control. Shown is the mean ± SD of three independent experiments. The red arrows mark the concentration at which PF-543, in the absence of SphK2 inhibitors, significantly reduced the amounts of S1P and dhS1P below solvent control level (see also supplemental Fig. S4). B: HK-2 SphK1 knockdown (kd) or vector control cells were stimulated with indicated concentrations of ABC294640 or K145 for 5 h. (Dh)S1P levels normalized to the solvent control were determined by LC-MS/MS. The mean ± SD of three independent experiments is shown. Statistically significant differences to the solvent control (bars at concentration “0”) were determined using two-way ANOVA with Šídák's test for multiple comparisons. ∗∗*P* < 0.01, ∗∗∗*P* < 0.001, ∗∗∗∗*P* < 0.0001.

In the further course of the study, we focused on the mechanistic elucidation of the unexpected increase of cellular S1P and dhS1P after treatment with the SphK2 inhibitors ABC294640 or K145. Assuming an effective inhibition of SphK2, our results could indicate possible (over)compensatory effects of SphK1. However, we did observe neither increased expression of either *SPHK1/2* mRNA, nor native or phosphorylated SphK1/2 protein after treatment with ABC294640 or K145 (data not shown). If SphK1 is the main cause of the enormous increase in (dh)S1P, costimulation of cells with ABC294640 or K145 and a potent SphK1 inhibitor should reverse these effects. We therefore treated Chang cells with 50 μM ABC294640 or 10 μM K145 in the absence or presence of increasing concentrations of PF-543. Indeed, from a concentration of 0.01 μM PF-543 proved to be very effective on dhS1P and S1P levels, so that these were significantly reduced in Chang cells compared to the control (supplemental Fig. S4, second row). Remarkably, the ability of PF-543 to lower (dh)S1P levels was strongly attenuated in the presence of ABC294640 (Fig. 4A). More precisely, PF-543 concentrations of 0.5 μM or even 5 μM were necessary to significantly reduce cellular S1P (60% of solvent control, *P* = 0.0002) or dhS1P (31% of solvent control, *P* = 0.0001), respectively. The attenuating effect was less pronounced in the presence of K145. Nevertheless, at least 0.05 μM PF-543 was required to significantly reduce cellular S1P (81% of solvent control, *P* = 0.0005) and dhS1P (71% of solvent control, *P* < 0.0001) levels. The effect of PF-543 on (dh)S1P levels in Chang cells was thus 5–500-fold weaker in the presence of either of the two SphK2 inhibitors tested. Another striking finding of this costimulation experiment was the particularly pronounced drop in S1P and dhS1P when the PF-543 concentration was increased from 0.1 μM to 0.5 μM (Fig. 4A). Interestingly, the IC_50_ of PF-543 for SphK2 was reported to be in this range (0.36 μM; (50)).

### Genetic knockdown of SphK1 does not prevent the increased cellular (dh)S1P levels caused by ABC294640 and K145

Next, we investigated the effect of treating SphK1-deficient cells with ABC294640 or K145 on (dh)S1P levels. For this purpose, we incubated human proximal tubule epithelial cells (HK-2) with a SphK1 knockdown verified at the mRNA and protein level (supplemental Fig. S9) and their vector controls with increasing concentrations of ABC294640 or K145. Of note, HK-2 cells are the fourth cell line in our studies in which treatment with ABC294640 or K145 led to a dose-dependent increase in S1P and dhS1P (Fig. 4B, filled bars). Impressively, the inhibitor-mediated dose-dependent increase in S1P and dhS1P was also observed in SphK1-deficient HK-2 cells and to a comparable extent as in the vector controls (Fig. 4B, hatched bars). From our cell cotreatments with ABC294640 or K145 and PF-543 as well as our studies on SphK1-deficient cells, we therefore conclude the following: *1)* an overcompensation of SphK1 as the main cause for the increased content of S1P and dhS1P after cell treatment with ABC294640 or K145 can be excluded. *2)* If increased (dh)S1P levels are also observed in SphK1-deficient cells, SphK2 must have a significant residual activity despite being targeted with ABC294640 or K145. *3)* Increased cellular levels of S1P and dhS1P despite the presence of high concentrations of both SphK1 and SphK2 inhibitors, indicates activation of metabolic pathways that lead to an increase in the corresponding molecular precursors (salvage and de novo synthesis pathway).

### ABC294640 and K145 show negligible inhibition of recombinant human SphK2 in vitro

**Fig. 5:**
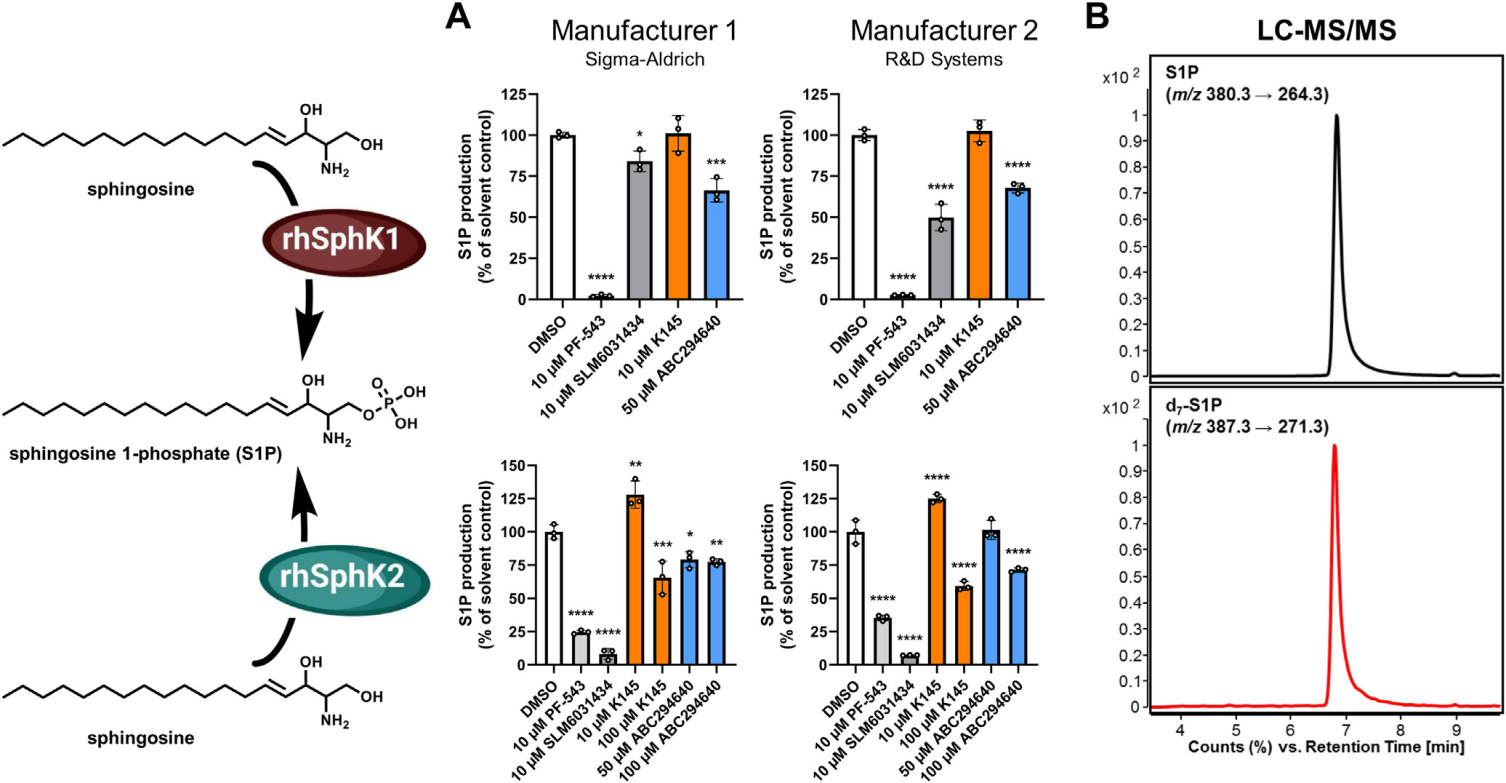
Effects of sphingosine kinase (SphK) inhibitors on the in vitro activity of recombinant human (rh) SphK1 and SphK2. A: The assay was performed with recombinant enzymes from two different companies, each incubated with 1 μM Sph and indicated concentrations of ABC294640, K145, PF-543 (positive control for SphK1), SLM6031434 (positive control for SphK2), or DMSO (solvent control) for 1 h at 37°C. Subsequently, the amounts of sphingosine 1-phosphate (S1P) formed were determined by LC-MS/MS. Shown is the mean ± SD of three independent experiments. Statistically significant differences compared to the solvent control (DMSO) were determined by one-way ANOVA with Dunnett's multiple comparison test. ∗*P* < 0.05, ∗∗*P* < 0.01, ∗∗∗*P* < 0.001, ∗∗∗∗*P* < 0.0001. B: LC-MS/MS chromatogram of the SphK product S1P and the internal standard d_7_-S1P used for accurate quantification.

Since (dh)S1P levels still increased when SphK1 and SphK2 were coinhibited, it is likely that the SphK2 inhibitors ABC294640 and K145 either only partially inhibit SphK2 or do not inhibit it at all. To investigate this hypothesis, we examined inhibition of recombinant human SphKs in a cell-free in vitro assay. For this purpose, we incubated either SphK1 or SphK2 protein, both purchased from two manufacturers (Sigma-Aldrich and R&D Systems), with Sph and analyzed the S1P production by LC-MS/MS (Fig. 5B). Of the three SphK2-specific inhibitors tested (K145, ABC294640, and SLM6031434), only K145 (10 μM) was found to be completely ineffective with respect to hSphK1 activity. ABC294640 (50 μM) reduced hSphK1-mediated S1P production by 32%–34% and SLM6031434 (10 μM) by 16%–50%. The positive control PF-543 (10 μM) was significantly more potent, reducing S1P formation by 97%–98% (Fig. 5A). Regarding hSphK2 activity, SLM6031434 (10 μM) as a positive control showed the expected strong inhibition, as the amount of S1P detected was less than 10% of that in the solvent control across protein sources. PF-543 at 10 μM also showed a pronounced inhibitory effect on hSphK2 and reduced the detectable S1P by 65%–75%. It is striking that the inhibitory effect of 50 μM ABC294640 or 10 μM K145 on hSphK2 was very low or absent. This was reproducible for proteins from both manufacturers. While K145 even caused a 25%–28% increase in hSphK2 activity, ABC294640 reduced S1P production only when the Sigma-Aldrich enzyme was used, and then only by 21%. When the R&D Systems protein was used, ABC294640 was completely ineffective. Weak inhibitory effects of both inhibitors on the two recombinant hSphK2 proteins used could only be observed when the concentrations of K145 or ABC294640 were increased tenfold or doubled, respectively. We therefore conclude that the maximum concentrations of ABC294640 and K145 used in the cell culture experiments are practically ineffective in the cell-free hSphK2 activity assay and, in the case of K145, even led to a moderate increase in SphK2 activity.

### ABC294640 and K145 affect sphingolipid de novo synthesis

**Fig. 6:**
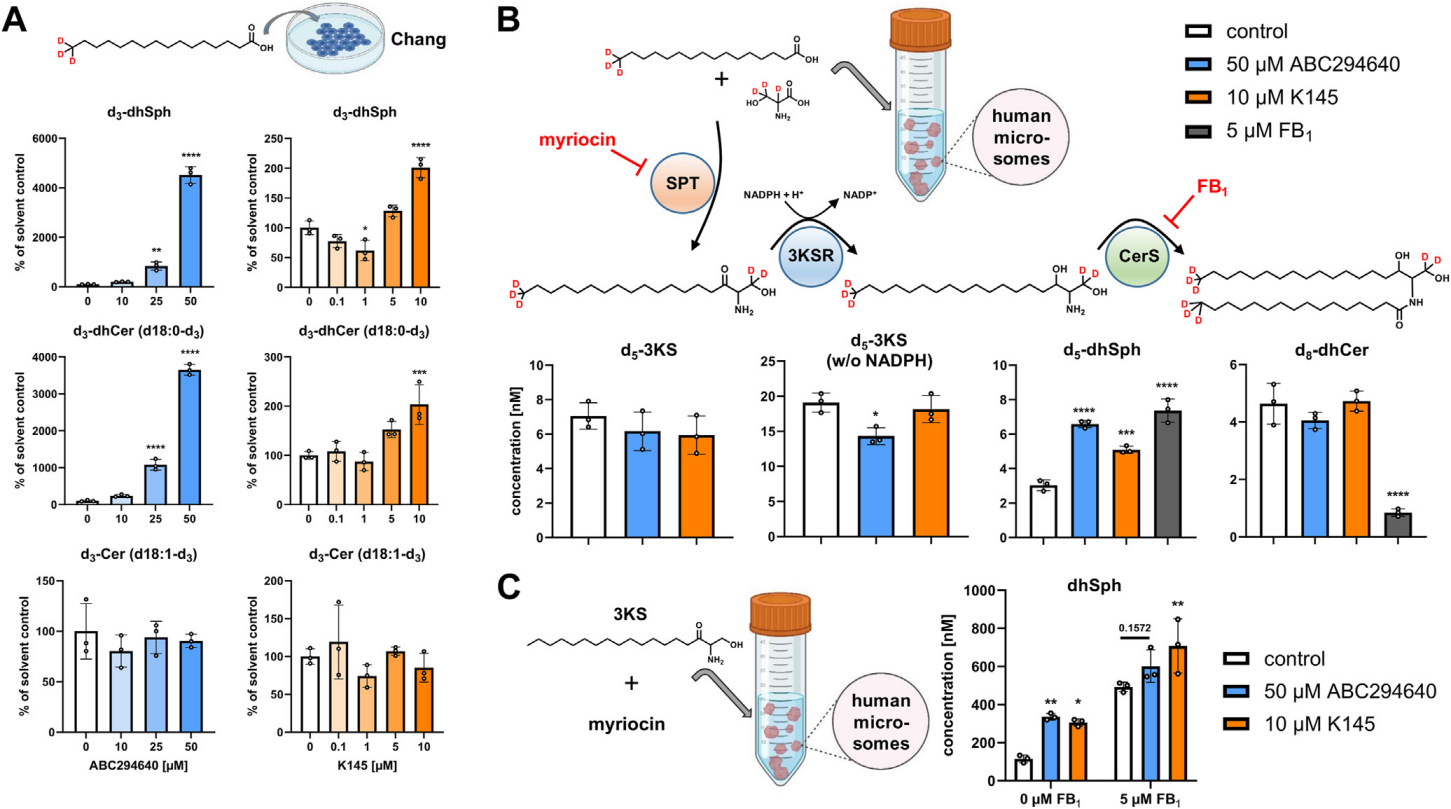
Impact of ABC294640 and K145 on sphingolipid de novo synthesis. A: De novo synthesis assay in Chang cells stimulated for 5 h with 10 μM d_3_-palmitate and the indicated inhibitor concentrations. Deuterated metabolites were analyzed by LC-MS/MS, and the amounts are presented relative to the solvent control (white bars). Dual incorporation of d_3_-palmitate also yielded d_6_-C16:0 Cer and dhCer species, which are included in the data presented. Shown is the mean ± SD of three independent experiments. B: Microsomal de novo synthesis assay, performed with 50 μM ABC294640, 10 μM K145 or 5 μM fumonisin B_1_ (FB_1_) for 1 h at 37°C. De novo formation of d_5_-3-ketodihydrosphingosine (d_5_-3KS), d_5_-dihydrosphingosine (d_5_-dhSph) and d_8_-C16:0 dihydroceramide (d_8_-dhCer) from d_3_-palmitate and d_3_-serine (loss of one deuterium label during SPT catalysis) was monitored by LC-MS/MS. Absolute concentrations in the lipid extract were calculated. Shown is the mean ± SD of three independent experiments. C: Modified version of the microsomal de novo synthesis assay in which serine palmitoyltransferase (SPT) was inhibited by 50 μM myriocin and 1 μM 3KS was used as substrate together with 50 μM ABC294640 or 10 μM K145 in the absence or presence of 5 μM FB_1_. Absolute concentrations of dhSph in the lipid extracts were determined by LC-MS/MS. Shown is the mean ± SD of three independent experiments. A and B: Statistically significant differences to the solvent controls (white bars) were determined using one-way ANOVA with Dunnett's multiple comparison test. ∗*P* < 0.05, ∗∗*P* < 0.01, ∗∗∗*P* < 0.001, ∗∗∗∗*P* < 0.0001. C: Statistically significant differences to the solvent control (white bars) were determined using two-way ANOVA with Šídák's test for multiple comparisons. ∗*P* < 0.05, ∗∗*P* < 0.01. 3KSR, 3-ketodihydrosphingosine reductase; Cer, ceramide; CerS, ceramide synthase.

Since the data presented so far indicate that the SphK2 inhibitors ABC294640 and K145 do not have an inhibitory effect on (dh)S1P levels, but actually increase them (Figs. 2 and 4; supplemental Figs. S4–S6), we have now addressed the question of possible underlying mechanisms. DhS1P can only be formed by SphK-mediated phosphorylation of dhSph (Fig. 1). Interestingly, increased levels of dhSph were measured for treatment with ABC294640 (in Chang, HepG2, and HUVEC cells) and with K145 (in HUVEC cells). For this reason, in the following experiment we investigated whether activation of de novo synthesis takes place and therefore we incubated Chang cells for 1 h with increasing concentrations of ABC294640 or K145, and then added 10 μM d_3_-palmitate for further 4 h. The chosen concentration of deuterated palmitate and the duration of stimulation had no influence on intrinsic SL homeostasis. However, it should be noted that d_3_-palmitate concentrations exceeding 50 μM led to decreases in selected intrinsic SL species (trendwise for dhS1P and significant for dhCer) (supplemental Fig. S10). Analysis of SL species bearing a d_3_-label in the sphingoid backbone (d18:0-d_3_ for dhSph and dhCer, d18:1-d_3_ for Cer) confirmed the significant modulation of de novo synthesis by both test compounds, but especially by ABC294640 (Fig. 6A). The latter, massively increased the levels of d_3_-dhSph and the subsequent metabolite d_3_-dhCer starting at a concentration of 25 μM. d_3_-Cer levels remained unchanged. K145 showed similar but much less pronounced effects. While 50 μM ABC294640 increased d_3_-dhSph and d_3_-dhCer levels 45-fold and 37-fold, respectively, there was a doubling of both labeled dhSph and dhCer after stimulation with 10 μM K145. Next, we aimed to identify the targets of ABC294640 and K145 within the de novo synthesis using our recently described microsomal de novo synthesis assay (62). Briefly, human liver microsomes served as source of ER-bound enzymes, thus including SPT, 3KSR, CerS, and DEGS (Fig. 1), and d_3_-palmitate and d_3_-l-serine as labeled SL precursors. We observed no appreciable difference in the formation of d_5_-3KS in the presence of ABC294640 (50 μM) or K145 (10 μM) compared to the solvent control (Fig. 6B). This was also the case when the assay was performed without addition of NADPH, which stops the de novo synthesis after the SPT reaction and therefore maximizes the d_5_-3KS yield. Consistent with the data from the cell experiments (Figs. 2 and 6A), we detected significantly increased amounts of d_5_-dhSph after incubation with ABC294640 or K145. Although different concentrations were used, the effects of ABC294640 and K145 were comparable to those of 5 μM FB_1_, an established CerS inhibitor known to increase dhSph levels. In contrast, completely different effects compared to FB_1_ were revealed after quantification of d_8_-dhCer. While FB_1_ reduced the formation of d_8_-dhCer to below 20% as expected, the levels detected after incubation with ABC294640 or K145 were not different from the solvent control. In an additional experiment, we then investigated the isolated 3KSR activity. In short, we incubated microsomes with the potent SPT inhibitor myriocin, used 3KS as assay substrate and stimulated with the indicated inhibitors. We analyzed the influence of 50 μM ABC294640 or 10 μM K145 on the formation of dhSph in the absence or presence of 5 μM FB_1_. As shown in Fig. 6C, ABC294640 and K145 again led to increased dhSph concentrations in the absence of FB_1_. As expected, the presence of FB_1_ increased the overall measurable dhSph concentration. Remarkably, even in the presence of FB_1_, the detectable amount of dhSph was still elevated compared to the solvent control, significant for K145 and borderline significant for ABC294640. From our studies with stable isotope-labeled SL precursors, we draw the following conclusion: *1)* both ABC294640 and K145 exert a significant influence on de novo synthesis, as evidenced by elevated levels of dhSph and dhCer. *2)* inhibition of DEGS by ABC294640 and K145 (see Fig. 3) is considered the main cause of increased dhCer levels. *3)* increased activity of 3KSR (but not SPT) contributes to increased levels of dhSph. *4)* stimulation of the de novo synthesis pathway in concert with virtually unchanged activities of both SphK isoforms after treatment with ABC294640 or K145 lead to massive increases in cellular dhS1P.

## Discussion

We noted that despite the growing evidence for off-target effects of prominent inhibitors of SL-metabolizing enzymes, most studies use them without monitoring potential unexpected effects on the sphingolipidome, e.g., by lipidomics. In order to close possible knowledge gaps and to contribute to a better understanding and interpretation of research data, we have aimed to elucidate further off-target effects of SphK inhibitors. We selected three cell lines commonly used as infection models, namely, Chang, HepG2, and HUVEC cells, and investigated the effects of seven SphK inhibitors on levels of selected cellular SLs. Four out of seven inhibitors (DMS, PF-543, SKI-II, and SLM6031434) showed the expected effects on S1P and dhS1P levels. However, the extent of the effects was different between the cell lines. HUVEC cells, for example, characterized by comparatively high basal levels of LCBs, were particularly sensitive to the presence of these four inhibitors. For instance, PF-543, the most potent inhibitor in our studies when applied at 10 μM, reduced S1P levels in HUVEC by 99%, while in HepG2 the levels were reduced by 87% and in Chang by 79%. Consequently, the weakest inhibitor in our studies, 5c, only showed a suppressive effect on S1P levels in the most sensitive HUVEC cell line (when applied at 10 μM), albeit only by 27%. Of note, 5c has already been used in the past without verifying the actual effect on the Sph-SphK-S1P axis (e.g., (48, 49)). For these cases, our results recommend at least considering alternative interpretations. If inhibitors have the opposite effect in the cell of what they were originally used for, there is a high risk of misinterpretation of research data. As far as we know, such findings for SphK inhibitors are not yet known in the literature. However, our inhibition study revealed such opposite effects for the SphK2-specific inhibitors ABC294640 and K145. Both consistently and significantly increased S1P and dhS1P levels in a total of four cell lines used. This is contrary to other studies. Liu *et al.* (56) were able to detect a significant reduction of S1P by 10 μM K145 after 3 h in U937 cells (human lung lymphoblasts) using LC-MS/MS. DhS1P was not measured. By means of sphingolipidomic analyses, Bennett *et al.* (64) detected a significant, approximately 25% S1P reduction in bortezomib-resistant 5TGM1.BR (myeloma) cells after 6 h-treatment with 8 μM K145. Again, dhS1P was not analyzed. By scintillation counting of [^3^H]S1P formed from applied [^3^H]Sph in HEK293T (human embryonic kidney) cells, Al-Shujairi *et al.* (65) reported a significant halving of S1P in the presence of 8 μM K145 after 30 min. However, the cells were simultaneously treated with 1 μM PF-543, which is known to inhibit both SphK isoforms in this concentration range (50). Numerous other studies used K145 without providing evidence of an actual (dh)S1P reduction (e.g., (66, 67, 68)). ABC294640 is currently the most extensively used SphK2 inhibitor in research, certainly aided by the fact that ABC294640 is the most advanced SphK inhibitor in clinical testing to date. It was investigated in a global phase 2/3 study in hospitalized patients with severe COVID-19 pneumonia (https://classic.clinicaltrials.gov/show/NCT04467840). Furthermore, ABC294640 was tested in a single-arm phase 2a clinical trial in patients with advanced, unresectable cholangiocarcinoma (https://classic.clinicaltrials.gov/show/NCT03414489) and in an investigator-sponsored phase 2 trial to evaluate its safety and efficacy in metastatic castration-resistant prostate cancer (69). Studies analyzing cellular SLs when using ABC294640 are scarce. In some studies, suppressed cellular S1P levels following ABC294640 incubation could be detected in radioactive (e.g., (70)) or immunological (e.g., (71)) assays. Weigert *et al.* (72) demonstrated by LC-MS/MS that cellular S1P levels in human primary macrophages were reduced after 24 h of stimulation with 10 μM ABC294640. In another recent study, 12.5 μM ABC294640 was able to reduce S1P levels in vemurafenib-resistant RKO (human colon carcinoma) cells only in combination with 7.5 μM vemurafenib (for 48 h), but not alone, as demonstrated by LC-MS/MS (73). None of the aforementioned studies showed data on dhS1P levels. An interesting observation was made by Alsanafi *et al.*, whose lipidomics data showed a slight increase in S1P (estimated 50%) after incubation of HEK293T cells with 25 μM ABC294640 for 24 h. Sph and dhSph were also increased, whereas dhS1P was decreased (74). To the best of our knowledge, this is the only published evidence so far of an unexpected opposite effect of ABC294640 on cellular S1P. We present here for the first time a dose-dependent S1P increase in four cell lines (Chang, HepG2, HUVEC, and HK-2 cells) after 5 h of incubation with ABC294640. The increases (given in parentheses as % of solvent control) were dramatic and reached statistical significance from a concentration of 10 μM in Chang (301%) and HUVEC (217%), 25 μM in HepG2 (228%), and 50 μM in HK-2 cells (546%). The highest inhibitor concentrations increased S1P 5.2–7.3-fold across cell lines. We also report for the first time a dose-dependent increase in dhS1P following cell stimulation with ABC294640. This accumulation was even more impressive, as the highest assay concentrations increased dhS1P by 5.9–338-fold. We would like to emphasize this finding in particular, as dhS1P has been much less intensively researched than S1P, but is just as capable of activating S1PRs and consequently causing various effects in the cell by triggering signaling cascades (75). We suspect a strong influence of cell type and incubation time on the effect of ABC294640 on sphingolipidoma. We could not find any study in the literature in which one of the cell lines we used was stimulated with ABC294640 alone and S1P was subsequently quantified. Furthermore, most published in vitro data of ABC294640 are based on cell stimulations of at least 24 h. In this study, however, data were obtained after 5 h of cell treatment, a duration that is more relevant for infection models, for example. Our findings are alarming because without corroborating research data by lipid quantification, ABC294640 effects could be attributed to S1P reduction that were actually caused by S1P elevation. Corresponding possible examples of this can already be found in the literature. While Schaper *et al.* (76) described an alleviating effect of topical S1P treatment in an imiquimod-induced psoriasis mouse model, Shin *et al.* reported an improvement of psoriasis in the same model by topical treatment with ABC294640, but not with PF-543. The authors of the latter study attributed the observed effects to reduced S1P formation due to SphK2 inhibition, but without measuring the S1P level (77).

It is known that SKI-II (35) and ABC294640 (63) increase cellular dhCer levels. In addition, increased Cer was detected for ABC294640, but not for SKI-II (74). Different mechanisms are discussed for both inhibitors. While SKI-II leads to increased polyubiquitination and thus proteasomal degradation of DEGS1, ABC294640 was not able to polyubiquitinate DEGS1 up to a concentration of 50 μM. A DEGS-dependent mechanism involving increased de novo biosynthesis of Cer is assumed for ABC294640 (54, 74). However, detailed insights are still lacking. We were able to confirm the findings for ABC294640 and SKI-II in our inhibition study. Both led to a strong increase in dhCer in Chang, HepG2, and HUVEC cells. Unlike SKI-II, ABC294640 also significantly elevated Cer contents in these cells. Interestingly, we also observed effects on the dhCer-Cer axis by all other inhibitors tested. To investigate the inhibitory effects of the test compounds in more detail and to exclude influences by cross-linked pathways (e.g., increased de novo synthesis of dhCer or degradation of dhSM), we used a cellular DEGS assay with deuterated C13:0 dhCer as substrate and LC-MS/MS-based monitoring of the conversion to deuterated C13:0 Cer. We observed an influence on DEGS activity for all inhibitors. With the exception of ABC294640 and SKI-II, this is a novel finding to the best of our knowledge. To date, a number of natural and synthetic substances have been identified that can inhibit the activity of DEGS (78). In addition to the two SphK inhibitors ABC294640 and SKI-II, for which this has been shown recently (74), our data suggest adding K145, PF-543, SLM6031434, and 5c to the panel of compounds with DEGS inhibiting properties. For SLM6031434 and DMS, increases in DEGS activity were also registered depending on the cell line. Molecular backgrounds of the newly discovered DEGS activity changes remain unanswered at this time. However, we strongly recommend further investigation of those mechanisms, especially for the SphK inhibitors PF-543 and SLM6031434, which are nowadays widely used due to their high potency and specificity. DhCer are no longer considered inert lipids. On the contrary, they are central to cell fate-determining processes such as autophagy (79) and play a crucial role in the pathogenesis of diseases, particularly of the central nervous system (80).

We further investigated how K145 and ABC294640 can lead to the described strong increases in cellular dhS1P and S1P. A general counter-regulation upon inhibition of SphK2 can be excluded, as the third SphK2 inhibitor tested, SLM6031434, reduced S1P and dhS1P in all cells in a dose-dependent manner. SphK1 is regulated by different mechanisms, e.g., at the transcriptional and translational level, by posttranslational modifications, by external stimuli or by interaction with other proteins and membranes (81). Neither for K145 nor for ABC294640 did we find evidence that gene expression or protein level was altered in the presence of either test substance (incubated for 5 h). For ABC294640, this contradicts the study by McNaughton *et al.* (54), who reported that 5–25 μM ABC294640 induces proteasomal degradation of SphK1, but after 24 h. Our lipidomics data also provide no indication of a SphK1 protein loss during the 5-h treatment with ABC294640 (50 μM) or K145 (10 μM), as the strong increase in S1P and dhS1P could be attenuated by stepwise titration with PF-543 (specific for SphK1). A particularly strong reduction in S1P and dhS1P occurred with the addition of 0.5 μM PF-543. In tests with recombinant enzymes, an IC_50_ of 2.7 nM for SphK1 and 356 nM for SphK2 was determined for PF-543 (50). It is therefore conceivable that in the presence of 500 nM PF-543 and above, both SphK isoforms were inhibited and consequently S1P and dhS1P levels decreased to a greater extent. This in turn suggests that 50 μM ABC294640 or 10 μM K145 did not sufficiently inhibit SphK2 in Chang cells. Our experiment with SphK1-deficient HK-2 cells provided further, even stronger evidence for this. Remarkably, S1P and dhS1P levels increased in the presence of 50 μM ABC294640 or 10 μM K145 to a comparable extent as in the vector control. Since SphK1 knockdown was verified at the mRNA and protein level, there must be considerable residual SphK2 activity in the presence of the inhibitors. During the development of K145, the IC_50_ for SphK2 was determined at 4.3 μM. No inhibition could be shown for SphK1 up to 10 μM. The corresponding assay was based on lysates of SphK1 or SphK2 overexpressing cells and a radiographic measurement of TLC-separated radiolabeled S1P (56). Inhibitory concentrations (IC_50_) of ABC294640 were determined on recombinant enzymes using NBD-Sph as substrate and HPLC separation with fluorescence detection of NBD-S1P. ABC294640 exhibited inhibition of SphK2 with an IC_50_ of approximately 60 μM without affecting the activity of SphK1 up to 100 μM (53). We wanted to reproduce these data and used an assay based on purified recombinant enzymes and stable isotope dilution LC-MS/MS for the detection of formed S1P. K145 did not inhibit SphK1 at a concentration of 10 μM, as described in the literature. Surprisingly, 10 μM K145 increased the activity of SphK2 by 25%–28%, which could contribute to the explanation of the increased cellular S1P and dhS1P levels we observed. At 100 μM K145 (toxic to all cell lines in our study), the predicted inhibition of SphK2 was detected, but only by 34%–41%. Our studies thus reveal a much weaker inhibitory effect of K145 on SphK2 than previously described. ABC294640 also proved to be a weaker SphK2 inhibitor than expected from literature data. At 50 μM the inhibition was 0%–21%, at 100 μM (toxic in cell culture) between 23% and 29%, thus not even close to IC_50_. Remarkably, 50 μM ABC294640 inhibited the activity of SphK1 by 32%–34%. Thus, our studies indicate that ABC294640 is a very weak SphK inhibitor overall, with a stronger effect on SphK1 than on SphK2, which contradicts the published data. For the interpretation of our results from cell culture experiments, we therefore assumed insufficient inhibition of both SphK isoforms when using 50 μM ABC294640 or 10 μM K145.

In the only study we are aware of, Alsanafi *et al.* (74) reported increased dhSph levels in HEK293T cells after incubation with 25 μM ABC294640 for 24 h. The authors therefore hypothesized an increased de novo synthesis in the presence of ABC294640, without investigating this further. In fact, dhSph can result from the 3KSR-mediated reduction of 3KS or from the ceramidase (CDase)-mediated deacylation of dhCer (Fig. 1). We also found dose dependently increased dhSph levels in all cell lines after ABC294640 administration and in HUVEC cells also for K145, which represents a new finding. Furthermore, in contrast to the above-mentioned study, we found a dose-dependent increase in dhS1P levels after stimulation with ABC294640 or K145. This supports the assumption of stimulation of de novo synthesis in conjunction with insufficient SphK inhibition that we discussed above. We therefore thoroughly investigated the influence of both compounds on de novo synthesis. First, we treated Chang cells with the labeled SL precursor d_3_-palmitate and monitored metabolic flux through de novo synthesis using LC-MS/MS, a versatile tool as recently reviewed (82). We were able to confirm the results of the initial inhibition studies: ABC294640 and K145 significantly increased de novo formed (d_3_-labeled) dhSph and dhCer. However, d_3_-Cer levels remained unchanged by both inhibitors, and we suspect the DEGS inhibition described above as the reason for this. While we already have evidence that inhibition of DEGS probably contributes substantially to the accumulation of dhCer, it remained to be clarified how the massive increases in dhSph occur. One possibility would be the cleavage of accumulated dhCer into dhSph catalyzed by CDases (Fig. 1). However, we did not investigate this aspect further in this study. On the other hand, increased formation via SPT and 3KSR and/or inhibition of CerS would be conceivable. To address this question, we used our recently described microsomal de novo synthesis assay, which allows modulation of the individual steps of this pathway by addition or omission of cofactors and inhibitors (62). We obtained no evidence that ABC294640 or K145 stimulated the SPT reaction. Consistent with the cellular metabolic flux study, we found significantly increased levels of d_5_-dhSph after incubation with ABC294640 or K145. We observed comparable effects with FB_1_, a well-established CerS inhibitor known to accumulate dhSph (83). However, the fact that ABC294640 and K145 do not inhibit CerS was shown by the unchanged d_8_-dhCer levels, which decreased drastically with FB_1_. This already indicated an increased activity of the 3KSR. To test this theory, we performed the microsomal assay with 3KS as substrate (instead of d_3_-palmitate and d_3_-serine) and inhibited both SPT with myriocin and CerS with FB_1_. Still, ABC294640 and K145 increased dhSph above control levels. We therefore assume that an increased activity of the 3KSR must be responsible for the accumulation of dhSph. This new insight is indeed surprising. 3KS is considered a highly transient SL intermediate that is converted to dhSph by the 3KSR at a high rate, suggesting negligible regulation at the 3KSR level. Consequently, 3KSR is one of the least studied and understood enzymes in SL metabolism. However, scientific interest in 3KSR appears to be growing. Two recent studies suggest that 3KSR plays a critical role in cancer cell fate by eliminating cytotoxic 3KS when SPT activity is elevated, e.g., in breast cancer (84), and maintaining ER homeostasis and the response to unfolded proteins in leukemia (85). An antitumor effect was observed for both ABC294640 (86) and K145 (56). We therefore believe that further mechanistic studies on the observed effects of these compounds on 3KSR activity are necessary and worthwhile.

**Fig. 7:**
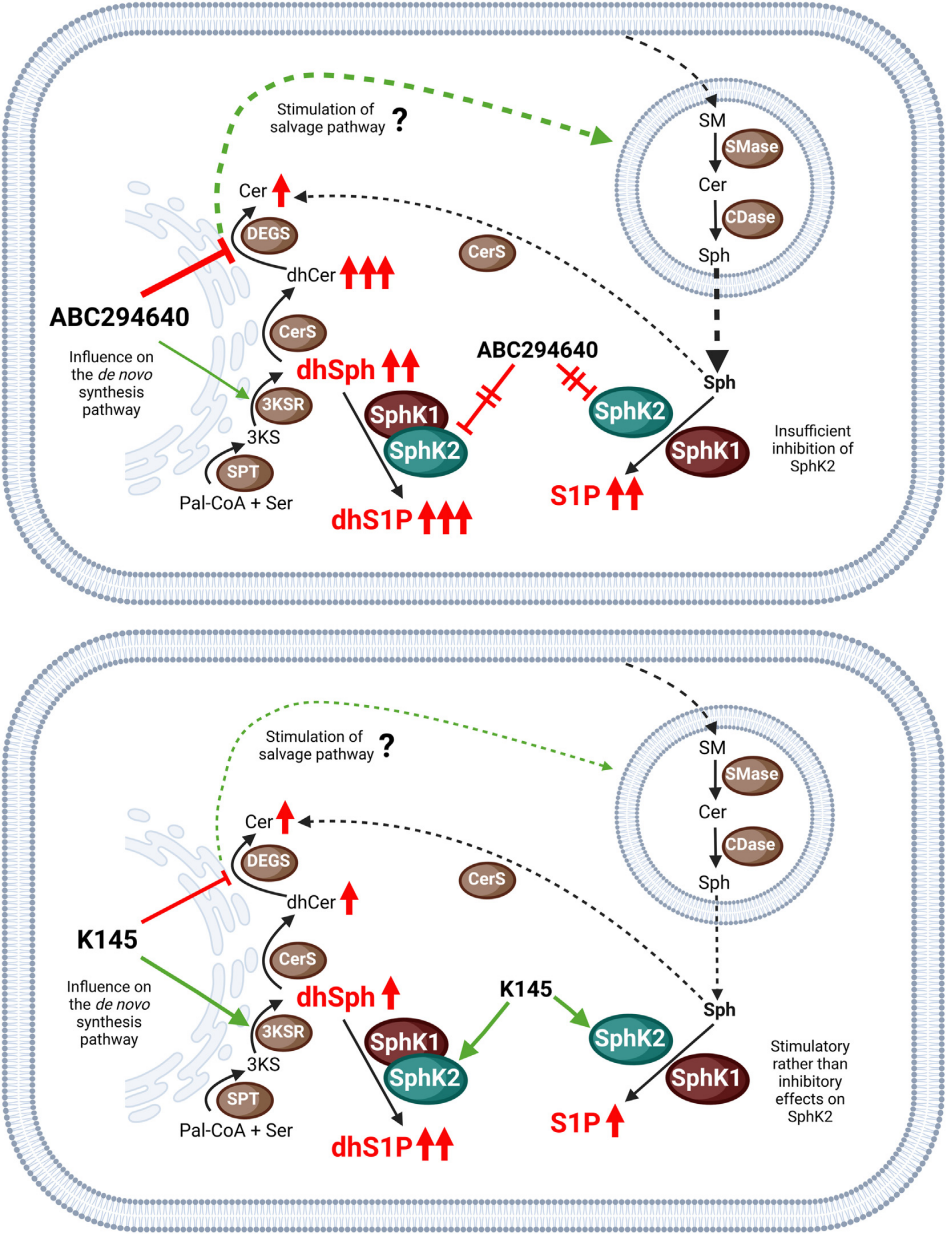
Proposed mechanisms of increased cellular S1P and dhS1P levels caused by ABC294640 (top panel) or K145 (bottom panel). Please note that either the line thickness or the number of arrows indicate the intensity of the observed effect. Green arrows indicate stimulating/activating effects. 3KS, 3-ketodihydrosphingosine; 3KSR, 3-ketodihydrosphingosine reductase; CDase, ceramidase; Cer, ceramide; CerS, ceramide synthase; DEGS, dihydroceramide desaturase; dhCer, dihydroceramide; dhS1P, dihydrosphingosine 1-phosphate; dhSph, dihydrosphingosine; Pal-CoA, palmitoyl-coenzyme A; S1P, sphingosine 1-phosphate; Ser, l-serine; SM, sphingomyelin; SMase, sphingomyelinase; SphK, sphingosine kinase; SPT, serine palmitoyltransferase.

Guan *et al.* showed an increase in Cer levels along with a decrease in S1P in A549 human primary lung cancer cells after 24 h of incubation with ABC294640 (up to 30 μM). The authors attributed the increasing Cer contents to a possible inhibition of glucosylceramide synthase by ABC294640 (87). However, clear evidence that ABC294640 is a functional inhibitor of this enzyme is not yet available in the literature. For cell stimulations with ABC294640, we observed both increasing S1P and Cer levels with concomitant increasing dhCer (Fig. 2). In addition, ABC294640 reduced cellular SM levels (supplemental Fig. S8). We therefore hypothesize that ABC294640 (and to a lesser extent probably K145) may stimulate the salvage pathway, possibly triggered by DEGS inhibition. However, data on glycosphingolipids are required to support this hypothesis, which we do not present in this study. Another branch of complex SL metabolism that was not investigated in our study is a possible effect of ABC294640 and K145 on the activity of S1P lyase, which irreversibly cleaves S1P/dhS1P into phosphoethanolamine and hexadecenal/hexadecanal, respectively (Fig. 1). The examination of both aspects would help to explain the elevated (dh)S1P levels, a claim that should be supported by further studies with more extensive sphingolipidome analyses. For now, we postulate the following molecular mechanisms for the observed cellular effects of ABC294640 and K145 (summarized graphically in Fig. 7): ABC294640 and K145 affect de novo synthesis by apparently increasing 3KSR activity and inhibiting DEGS. Both lead to an accumulation of dhSph, which is more pronounced with ABC294640, presumably due to its more effective DEGS inhibition. Via a mechanism yet to be elucidated, ABC294640 and to a lesser extent probably also K145 may stimulate the salvage pathway and thus increase Sph recycling. Both SphK isoforms are very likely almost unchanged in their activity in the presence of the two compounds. In the case of K145, there is even evidence of a slight increase in SphK2 activity. Together with the accumulation of SphK substrates, this leads to an increase in dhS1P and S1P (strong for ABC294640 and moderate for K145).

In conclusion, our study points to the urgency of monitoring the cellular sphingolipidome when inhibitors of SL-metabolizing enzymes are used and mechanistic conclusions are drawn. None of the seven SphK inhibitors we tested was free of unexpected on-target and/or off-target effects. For the inhibitors K145 and ABC294640, which are thought to be SphK2-specific, we dramatically observed the opposite of what might be expected in terms of SL levels. We therefore have reason to suspect that the use of these inhibitors without verification at the lipid level may have led to misinterpretations within numerous published studies. Our data collection is intended to raise awareness for cautious data interpretation when using enzyme inhibitors. Similarly, our study should be an incentive to perform similar studies with inhibitors for other SL-metabolizing enzymes.

## Data availability

Data used in this study are available in the article or from the corresponding author upon request.

## Supplemental data

This article contains supplemental data.

## Conflict of interest

The authors declare that they have no conflicts of interest with the contents of this article.
