# Supplementary material for: The sphingosine kinase 2 inhibitors ABC294640 and K145 elevate (dihydro)sphingosine 1-phosphate levels in various cells
Source: J Lipid Res. 2024 Aug 23;65(10):100631. doi: 10.1016/j.jlr.2024.100631 (PMC11465068; doi:10.1016/j.jlr.2024.100631)
Supplement: Supplemental Figures and Tables [file mmc1.pdf]

Supplemental material

The sphingosine kinase 2 inhibitors ABC294640 and K145 elevate (dihydro)sphingosine 1-phosphate levels in various cells

Agata Prell[1], Dominik Wigger[1], Andrea Huwiler[2], Fabian Schumacher[1], Burkhard Kleuser[1]

[1] Institute of Pharmacy, Department of Pharmacology and Toxicology, Freie Universität Berlin, Berlin, Germany

[2] Institute of Pharmacology, Inselspital, INO-F, University of Bern, Bern, Switzerland

Address for correspondence

Burkhard Kleuser

Department of Pharmacology and Toxicology, Institute of Pharmacy,

Freie Universität Berlin, Königin-Luise-Str. 2+4, 14195 Berlin, Germany

email: kleuser@zedat.fu-berlin.de

Table of content

Suppl. Table 1: Primer sequences for RT-qPCR experiments……………………………………………………… 3

Suppl. Table 2: LC gradient for sphingolipid separation………………………………………………………… 4

Suppl. Table 3: LC-MS/MS settings for assessment of cellular DEGS activity………………………………… 5

Suppl. Table 4: LC-MS/MS settings for quantification of sphingolipid subspecies…………………………….. 6

Suppl. Table 5: LC-MS/MS settings for monitoring the sphingolipid *de novo* synthesis……………………….. 8

Suppl. Figure 1: Gene expression of *SPHK1*, *SPHK2* and *DEGS1* in Chang, HepG2 and HUVEC cells……………………...9

Suppl. Figure 2: Sphingolipid profile of untreated Chang, HepG2 and HUVEC cells……………………………10

Suppl. Figure 3: MTT cell viability assays with SphK inhibitors in Chang, HepG2 and HUVEC cells……………………….11

Suppl. Figure 4: Effects of SphK inhibitors on LCB levels in Chang cells………………………………………… 13

Suppl. Figure 5: Effects of SphK inhibitors on LCB levels in HepG2 cells………………………………………...14

Suppl. Figure 6: Effects of SphK inhibitors on LCB levels in HUVEC cells……………………………………...15

Suppl. Figure 7: Effects of SphK inhibitors on dhCer and Cer levels in Chang, HepG2 and HUVEC cells…………………...16

Suppl. Figure 8: Effects of ABC294640 and K145 on dhCer, Cer, dhSM and SM subspecies in Chang cells……….……..17

Suppl. Figure 9: *SPHK1* and *SPHK2* gene and protein expression in SphK1-deficient HK-2 cells…………………………18

Suppl. Figure 10: Effect of d_3-palmitate incubation on intrinsic and *de novo* formed sphingolipid levels in Chang cells……..19

Suppl. Table 1: Oligonucleotide primer sequences and product sizes for RT-qPCR experiments.

Target Gene	Gene Accession Number	Sequence	Fragment Size (bp)
h-HMBS	NM_000190.3	fw: ACCAAGGAGCTTGAACATGC<br>rv: GAAAGACAACAGCATCATGAG	143
h-DEGS1	NM_001321541.2<br>NM_003676.4	fw: CAGCTAGTCTGCAAGCCAC<br>rv: CTCTGGATACTTTGCCAGGAT	125
h-SPHK1	NM_001142601.2	fw: CCTTCACGCTGATGCTCACT<br>rv: CGTTCACCACCTCGTGCAT	126
h-SPHK2	NM_001204159.3	fw: ACTGCCCTCACCTGTCTGCT<br>rv: GGCGTGGTTCTGTCGTTCTGT	216

Gene accession numbers refer to the GenBank® sequence database provided by the National Center for Biotechnology Information (NCBI, USA). HMBS, hydroxymethylbilane synthase; DEGS, dihydroceramide delta-4 desaturase; SPHK, sphingosine kinase; fw, forward; rv, reverse; bp, base pairs.

Suppl. Table 2: LC gradient for separation of sphingolipid subspecies.

Time [min]	Solvent A [%][a]	Solvent B [%][b]	Flow [mL/min]
0	40	60	0.5
7	10	90	0.5
8	6	94	0.7
26	2	98	0.7
27	2	98	1.0
29	2	98	1.0
30	40	60	0.5
35[c]	40	60	0.5

[a] water (0.1% formic acid)

[b] acetonitrile/methanol (1:1, 0.1% formic acid)

[c] post-run

Suppl. Table 3: LC-MS/MS settings for assessment of cellular DEGS activity.

Compound	Precursor ion (*m/z*)	Product ion (*m/z*)[a]	Collision energy (eV)	Fragmentor voltage (V)	Retention time (min)	ISTD	Calibration Reference Compound
d_7-C13:0 dhCer	505.5	**487.5**	16	150	12.3	C17 Cer	C16:0 Cer
		291.3	28				
		273.3	32				
d_7-C13:0 Cer	485.5	289.3	20	150	11.9	C17 Cer	C16:0 Cer
		**271.3**	20				
		259.2	20				
C16:0 Cer	520.5	282.2	20	180	14.1	C17 Cer	C16:0 Cer
		**264.2**	24				
C18:0 SM	731.6	**184.0**	24	190	15.3	d_{31}-C16 SM	-
		86.2	80				
C17:0 Cer	534.5	282.3	24	190	15.0	-	-
		**264.3**	24				
d_{31}-C16:0 SM	734.8	**184.2**	28	200	13.3	-	-
		85.9	80				

[a] Quantifier mass transitions are given in bold style. ISTD, internal standard.

Suppl. Table 4: LC-MS/MS settings for quantification of sphingolipid subspecies.

Compound	Precursor ion (*m/z*)			Product ion (*m/z*) [a]			Retention time (min)	ISTD	Calibration Reference Compound	
	canonical	deuterated (d_3)	deuterated (d_6)	canonical	deuterated (d_3)	deuterated (d_6)			canonical	deuterated (d_3, d_6)
Sph	300.3	303.3	-	**282.3 (8)** / 252.3 (16)	**285.3 (8)** / 255.3 (16)	-	5.5	d_7-Sph	-	-
dhSph	302.3	305.3	-	**284.3 (12)** / 254.3 (20)	**287.3 (12)** / 257.3 (20)	-	5.8	d_7-dhSph	-	-
S1P	380.3	383.3	-	**264.3 (20)** / 82.1 (32)	**267.3 (20)** / 82.1 (32)	-	6.8	d_7-S1P	-	-
dhS1P	382.3	385.3	-	**284.3 (12)** / 266.4 (16)	**287.3 (12)** / 269.4 (16)	-	7.2	d_7-S1P	-	-
C16:0 Cer	520.5	523.5	526.5	**264.3 (24)** / 282.3 (24)	**267.3 (24)** / 285.3 (24)	**267.3 (24)** / 285.3 (24)	13.7	C17:0 Cer	C16:0 Cer	C16:0 Cer
C18:0 Cer	548.5	551.5	-	**264.2 (24)** / 282.3 (28)	**267.2 (24)** / 285.3 (28)	-	15.6	C17:0 Cer	C18:0 Cer	C18:0 Cer
C20:0 Cer	576.6	579.6	-	**264.3 (32)** / 282.3 (28)	**267.3 (32)** / 285.3 (28)	-	18.0	C17:0 Cer	C20:0 Cer	C20:0 Cer
C22:0 Cer	604.6	607.6	-	**264.3 (34)** / 282.3 (30)	**267.3 (34)** / 285.3 (30)	-	21.0	C17:0 Cer	C22:0 Cer	C22:0 Cer
C24:0 Cer	632.6	635.6	-	**264.3 (36)** / 282.3 (28)	**267.3 (36)** / 285.3 (28)	-	24.5	C17:0 Cer	C24:0 Cer	C24:0 Cer
C24:1 Cer	630.6	633.6	-	**264.3 (36)** / 282.3 (32)	**267.3 (36)** / 285.3 (32)	-	21.2	C17:0 Cer	C24:1 Cer	C24:1 Cer
C16:0 dhCer	540.5	543.5	546.5	**522.6 (20)** / 284.3 (28)	**525.6 (20)** / 287.3 (28)	**528.6 (20)** / 287.3 (28)	14.2	C17:0 Cer	C16:0 dhCer	C16:0 dhCer
C18:0 dhCer	568.5	571.5	-	**550.5 (20)** / 284.3 (28)	**553.5 (20)** / 287.3 (28)	-	16.3	C17:0 Cer	C18:0 dhCer	C18:0 dhCer
C20:0 dhCer	596.6	599.6	-	**578.6 (22)** / 284.3 (32)	**581.6 (22)** / 287.3 (32)	-	18.9	C17:0 Cer	C18:0 dhCer	C18:0 dhCer
C22:0 dhCer	624.6	627.6	-	**606.6 (22)** / 284.3 (32)	**609.6 (22)** / 287.3 (32)	-	22.1	C17:0 Cer	C24:0 dhCer	C24:0 dhCer
C24:0 dhCer	652.7	655.7	-	**634.6 (24)** / 284.3 (36)	**637.6 (24)** / 287.3 (36)	-	25.7	C17:0 Cer	C24:0 dhCer	C24:0 dhCer
C24:1 dhCer	650.7	653.7	-	**632.7 (24)** / 284.3 (36)	**635.7 (24)** / 287.3 (36)	-	22.3	C17:0 Cer	C24:1 dhCer	C24:1 dhCer
C16:0 SM	703.6	706.6	709.6	**184.0 (8)** / 86.1 (76)	**184.0 (8)** / 86.1 (76)	**184.0 (8)** / 86.1 (76)	12.8	d_{31}-C16:0 SM	C16:0 SM	C16:0 SM
C18:0 SM	731.6	734.6	-	**184.0 (28)** / 86.1 (76)	**184.0 (28)** / 86.1 (76)	-	14.7	d_{31}-C16:0 SM	C18:0 SM	C18:0 SM
C20:0 SM	759.6	762.6	-	**184.0 (28)** / 86.1 (78)	**184.0 (28)** / 86.1 (78)	-	17.0	d_{31}-C16:0 SM	C20:0 SM	C20:0 SM
C22:0 SM	787.7	790.7	-	**184.0 (28)** / 86.1 (78)	**184.0 (28)** / 86.1 (78)	-	19.4	d_{31}-C16:0 SM	C22:0 SM	C22:0 SM
C24:0 SM	815.7	818.7	-	**184.0 (28)** / 86.1 (80)	**184.0 (28)** / 86.1 (80)	-	22.7	d_{31}-C16:0 SM	C24:0 SM	C24:0 SM
C24:1 SM	813.7	816.7	-	**184.0 (8)** / 86.1 (80)	**184.0 (8)** / 86.1 (80)	-	19.5	d_{31}-C16:0 SM	C24:1 SM	C24:1 SM
C16:0 dhSM	705.6	708.6	711.6	**184.0 (8)** / 86.1 (76)	**184.0 (8)** / 86.1 (76)	**184.0 (8)** / 86.1 (76)	13.5	d_{31}-C16:0 SM	C16:0 SM	C16:0 SM
C18:0 dhSM	733.6	736.6	-	**184.0 (28)** / 86.1 (76)	**184.0 (28)** / 86.1 (76)	-	15.6	d_{31}-C16:0 SM	C18:0 SM	C18:0 SM
C20:0 dhSM	761.6	764.6	-	**184.0 (28)** / 86.1 (78)	**184.0 (28)** / 86.1 (78)	-	18.0	d_{31}-C16:0 SM	C20:0 SM	C20:0 SM
C22:0 dhSM	789.7	792.7	-	**184.0 (28)** / 86.1 (78)	**184.0 (28)** / 86.1 (78)	-	20.9	d_{31}-C16:0 SM	C22:0 SM	C22:0 SM
C24:0 dhSM	817.7	820.7	-	**184.0 (28)** / 86.1 (80)	**184.0 (28)** / 86.1 (80)	-	24.5	d_{31}-C16:0 SM	C24:0 SM	C24:0 SM
C24:1 dhSM	815.7	818.7	-	**184.0 (8)** / 86.1 (80)	**184.0 (8)** / 86.1 (80)	-	20.9	d_{31}-C16:0 SM	C24:1 SM	C24:1 SM

Suppl. Table 4 (continued)

Compound	Precursor ion (*m/z*)			Product ion (*m/z*) [a]			Retention time (min)	ISTD	Calibration Reference Compound	
	canonical	deuterated (d_3)	deuterated (d_6)	canonical	deuterated (d_3)	deuterated (d_6)			canonical	deuterated (d_3, d_6)
d_7-Sph	307.3	-	-	**289.3 (8)** / 259.3 (20)	-	-	5.5	-	-	-
d_7-dhSph	309.4	-	-	**291.3 (12)** / 261.3 (24)	-	-	5.8	-	-	-
d_7-S1P	387.3	-	-	**271.3 (20)** / 82.1 (36)	-	-	6.8	-	-	-
C17:0 Cer	534.5	-	-	**264.3 (24)** / 282.3 (28)	-	-	14.6	-	-	-
d_{31}-C16:0 SM	734.6	-	-	**184.0 (28)** / 86.1 (76)	-	-	12.7	-	-	-

[a] Quantifier mass transitions are given in bold style. Collision energies (in eV) are given in parentheses. ISTD, internal standard.

Suppl. Table 5: LC-MS/MS settings for monitoring the sphingolipid *de novo* synthesis (microsomal assay)

Compound	Precursor ion (*m/z*)	Product ion (*m/z*)[a]	Collision energy (eV)	Retention time (min)	ISTD	Calibration Reference Compound
d_5-3KS	305.3	287.3	16	6.0	C17 dhSph	3KS
		**273.3**	16			
d_5-dhSph	307.3	**289.3**	12	5.9	C17 dhSph	dhSph
		257.3	20			
d_8-C16:0 dhCer	548.6	**530.6**	20	14.2	C17:0 Cer	C16:0 dhCer
		289.3	28			
3KS	300.3	282.3	16	6.0	C17 dhSph	3KS
		**270.3**	16			
dhSph	302.3	**284.3**	12	5.9	C17 dhSph	dhSph
		254.3	20			
C16:0 dhCer	540.5	**522.6**	20	14.2	C17:0 Cer	C16:0 dhCer
		284.3	28			
C17 dhSph	288.3	**270.3**	12	5.3	-	-
		240.2	20			
C17:0 Cer	534.5	282.3	28	14.6	-	-
		**264.3**	24			

[a] Quantifier mass transitions are given in bold style. ISTD, internal standard.

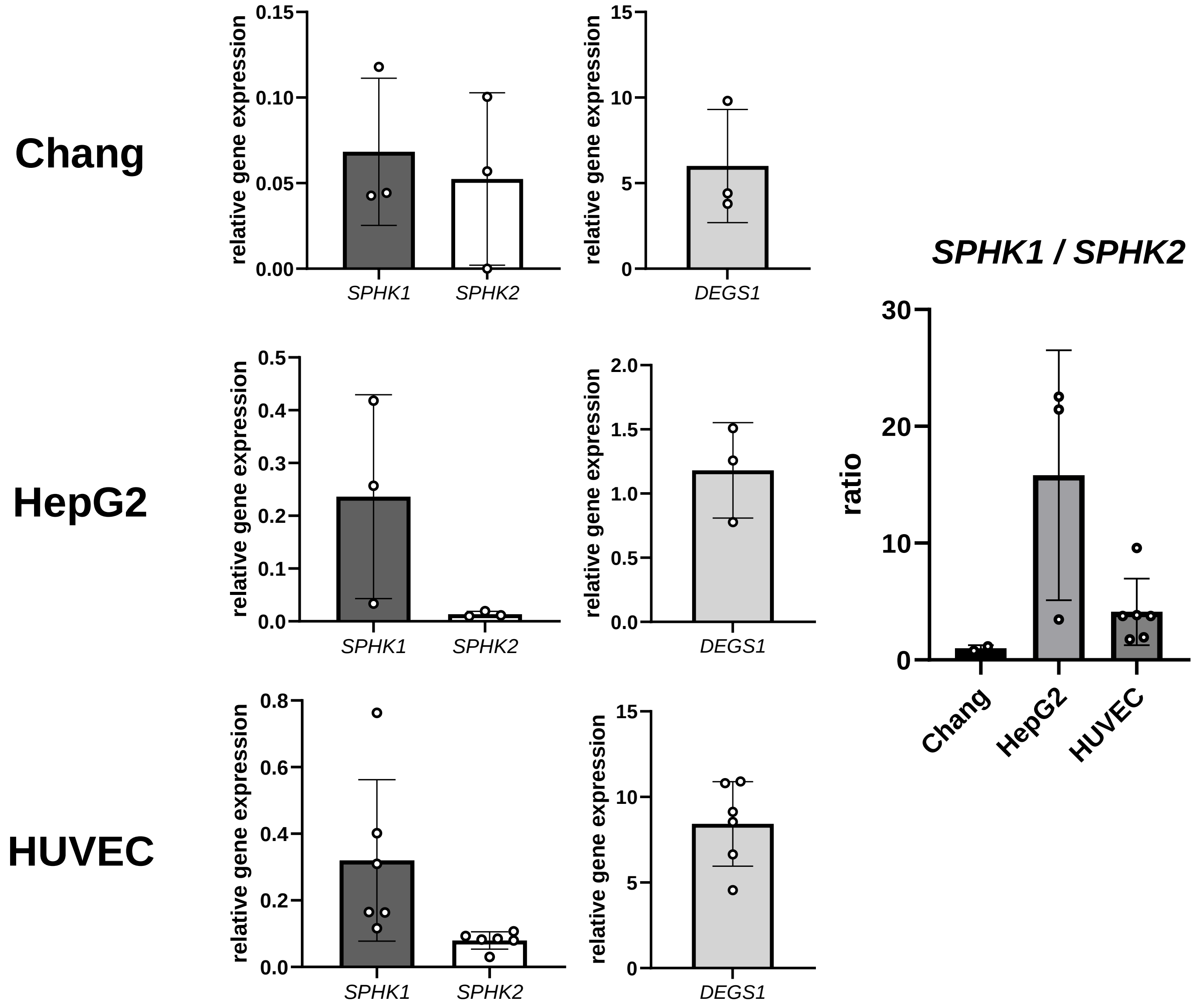

Suppl. Figure 1. Relative gene expression of sphingosine kinase 1 (*SPHK1*) and 2 (*SPHK2*), and dihydroceramide desaturase (*DEGS1*) in Chang, HepG2 and HUVEC cells determined by qPCR. *HMBS* was used as housekeeping gene. Shown is the mean ± SD of 3-6 independent experiments.

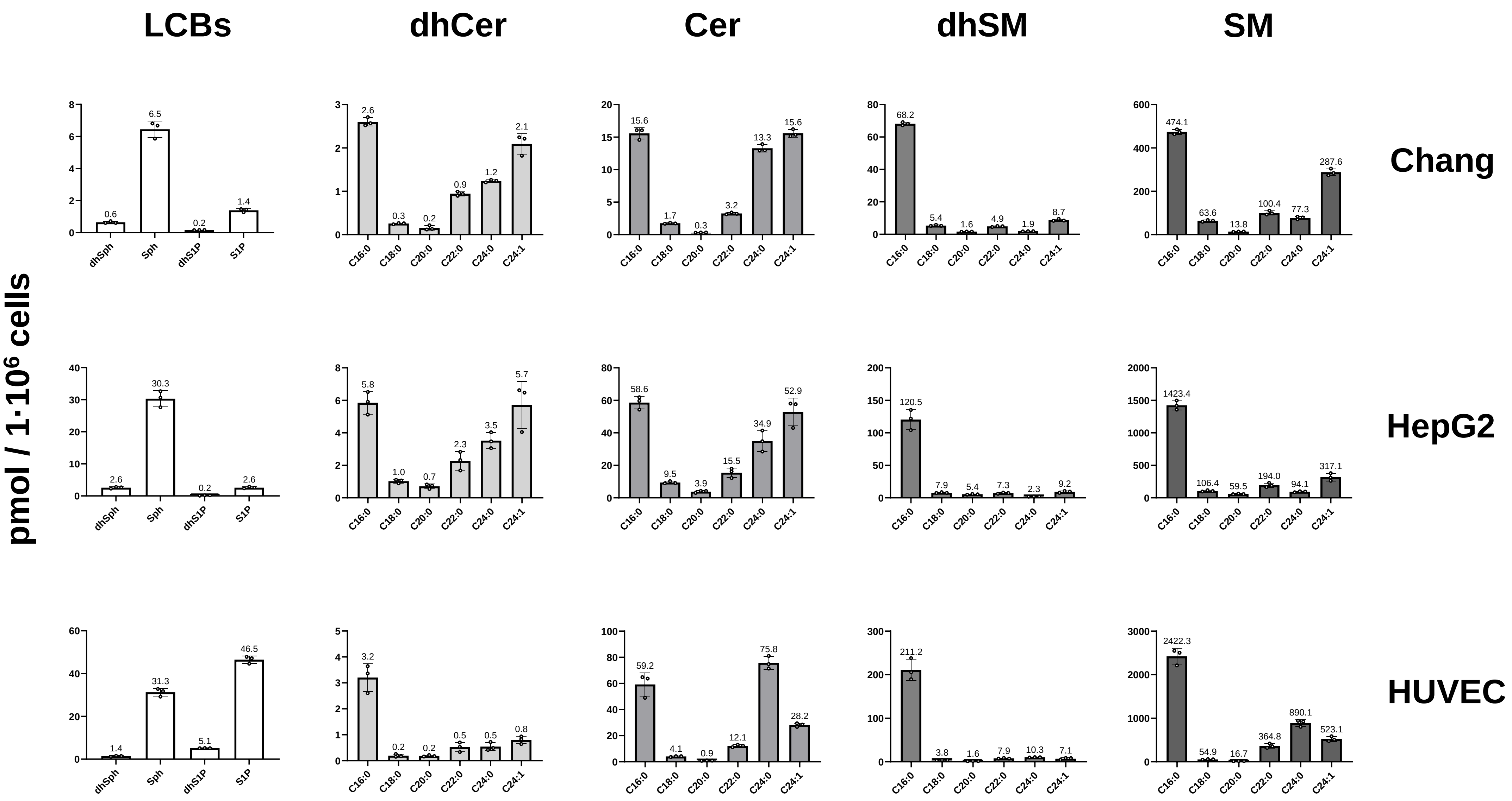

Suppl. Figure 2. Sphingolipid profile of untreated Chang, HepG2 and HUVEC cells determined by LC-MS/MS. The amounts were normalized to cell number. Shown is the mean ± SD of 3 independent experiments. The mean is also displayed above the bars for better visibility of small values. Cer, ceramide; dhCer, dihydroceramide; dhS1P, dihydrosphingosine 1-phosphate; dhSM, dihydrosphingomyelin; dhSph, dihydrosphingosine; LCBs, long-chain bases; S1P, sphingosine 1-phosphate; SM, sphingomyelin; Sph, sphingosine.

MTT assay (24 h)

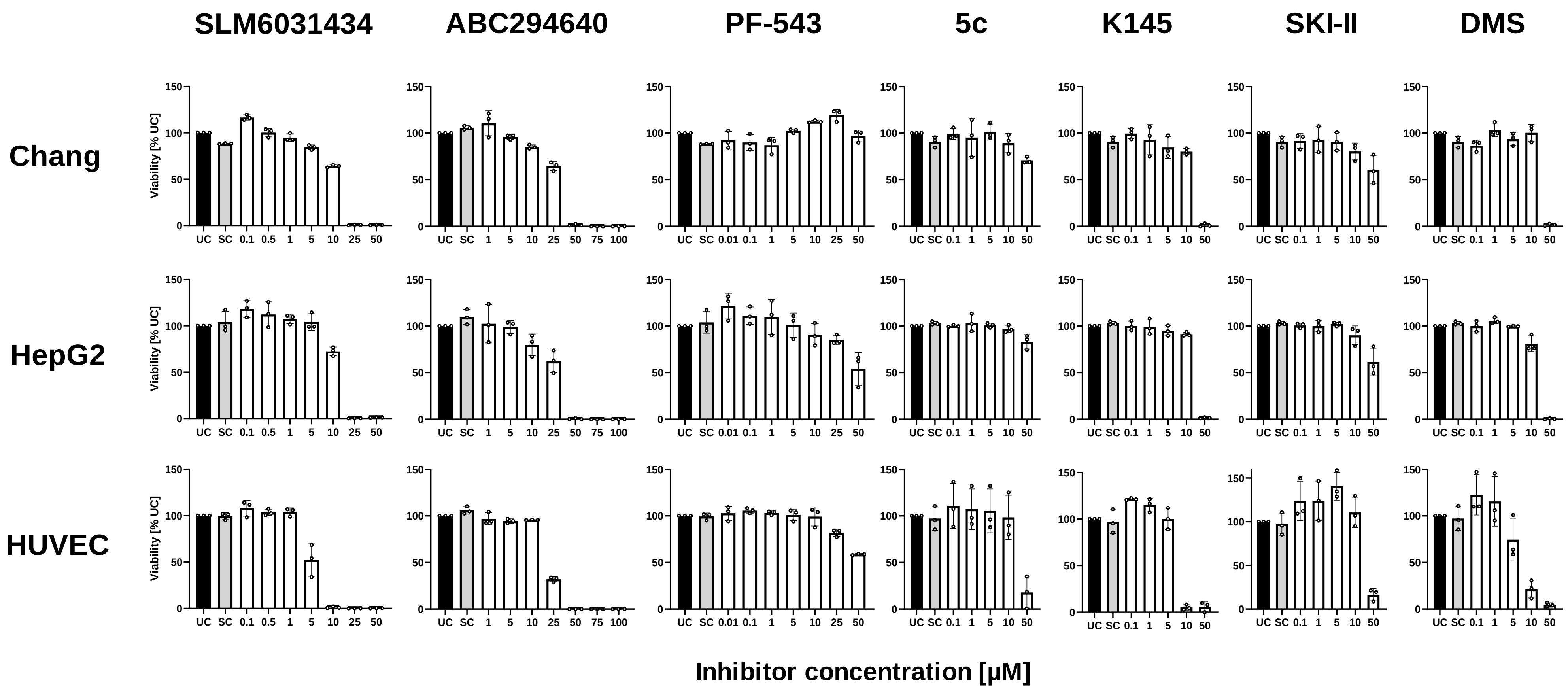

Suppl. Figure 3. MTT cell viability assays performed in Chang, HepG2 and HUVEC cells with indicated concentrations of sphingosine kinase inhibitors and incubation duration of 24 h. A cell viability <75% predicts cytotoxic effects. Shown is the mean ± SD of 3 independent experiments. UC, untreated control; SC, solvent control.

MTT assay (5 h)

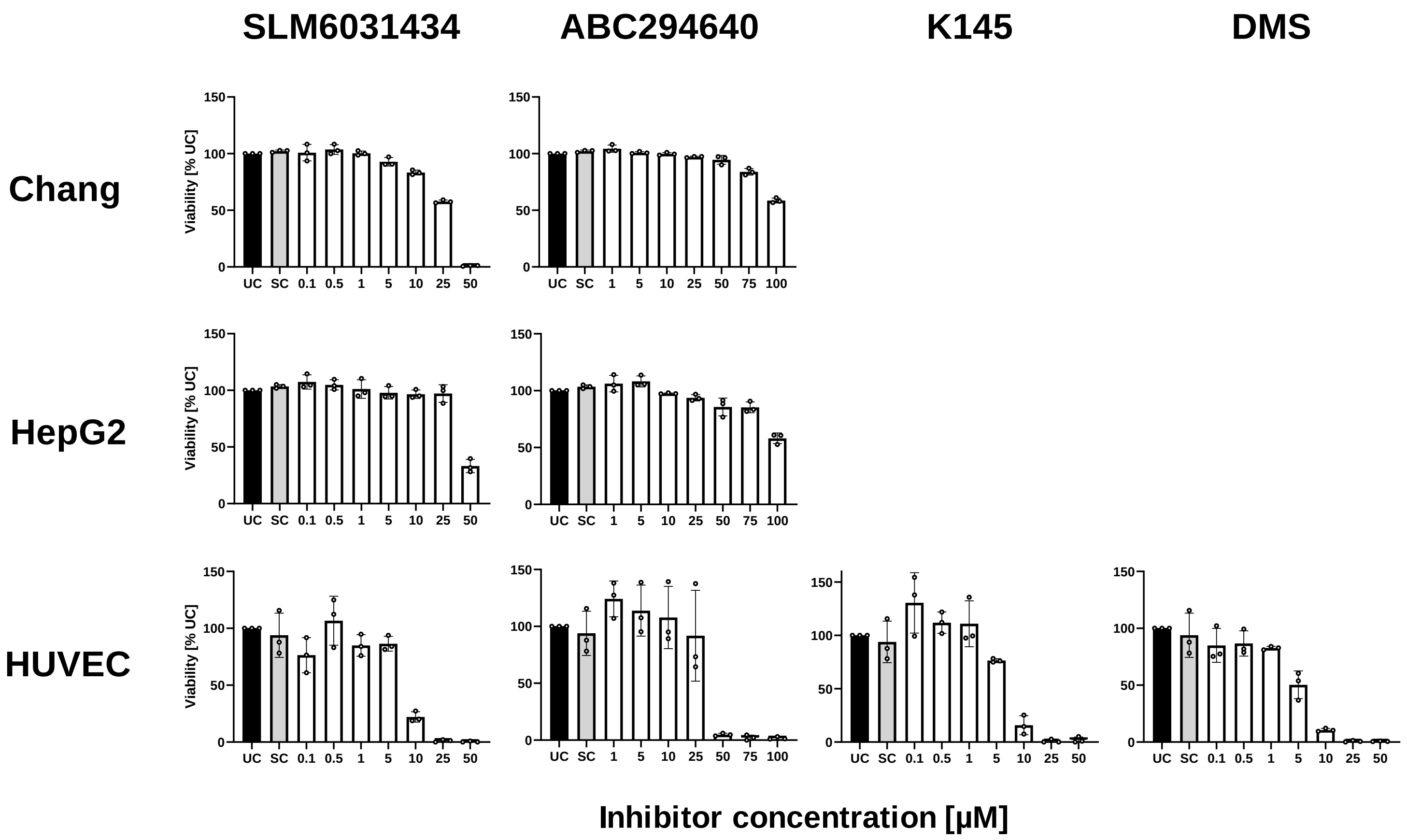

Suppl. Figure 3 (continued). MTT cell viability assays performed in Chang, HepG2 and HUVEC cells with indicated concentrations of sphingosine kinase inhibitors and incubation duration of 5 h. A cell viability <75% predicts cytotoxic effects. Shown is the mean ± SD of 3 independent experiments. UC, untreated control; SC, solvent control.

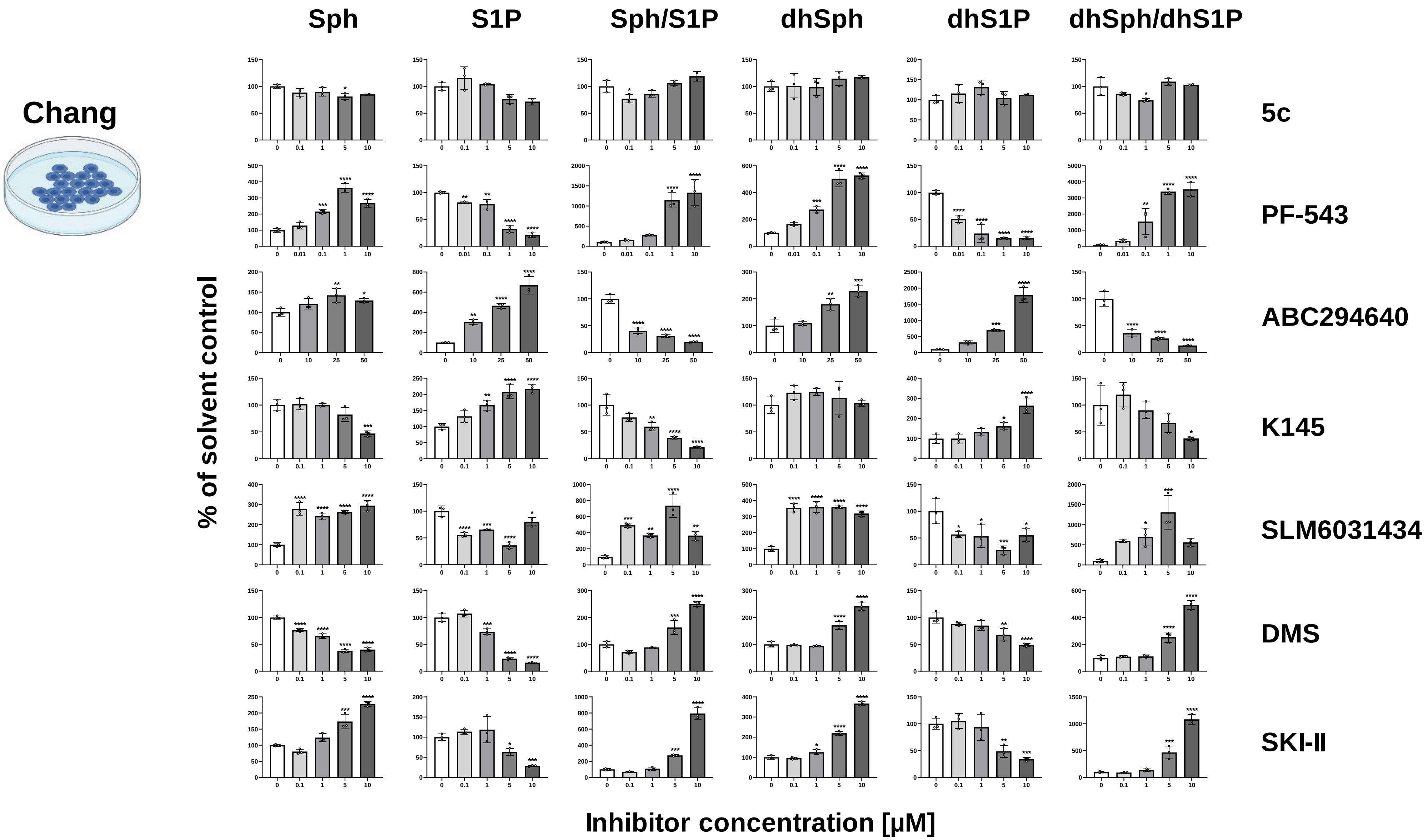

Suppl. Figure 4. Effects of sphingosine kinase inhibitors on the content of sphingosine (Sph), sphingosine 1-phosphate (S1P), dihydrosphingosine (dhSph), dihydrosphingosine 1-phosphate (dhS1P) and selected ratios thereof in Chang cells, determined by LC-MS/MS. Cells were treated for 5 h with the indicated concentrations of the inhibitors and quantified amounts were normalized to the solvent control. Shown is the mean ± SD of 3 independent experiments. Statistically significant differences to the solvent control (white bars) were determined using one-way ANOVA with Dunnett's multiple comparison test. *$p<0.05$, **$p<0.01$, ***$p<0.001$, ****$p<0.0001$.

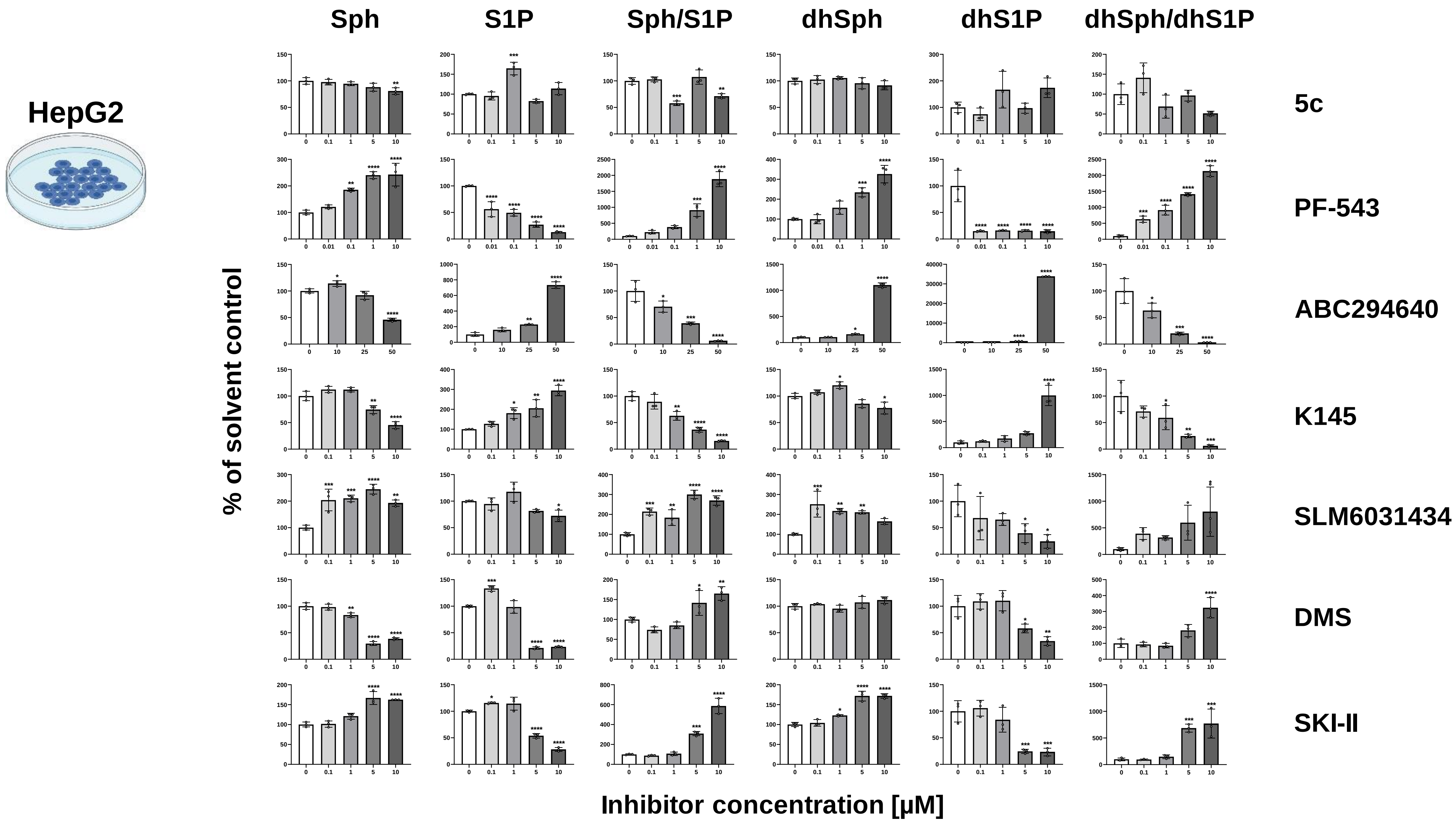

Suppl. Figure 5. Effects of sphingosine kinase inhibitors on the content of sphingosine (Sph), sphingosine 1-phosphate (S1P), dihydrosphingosine (dhSph), dihydrosphingosine 1-phosphate (dhS1P) and selected ratios thereof in HepG2 cells, determined by LC-MS/MS. Cells were treated for 5 h with the indicated concentrations of the inhibitors and quantified amounts were normalized to the solvent control. Shown is the mean ± SD of 3 independent experiments. Statistically significant differences to the solvent control (white bars) were determined using one-way ANOVA with Dunnett's multiple comparison test. *p<0.05, **p<0.01, ***p<0.001, ****p<0.0001.

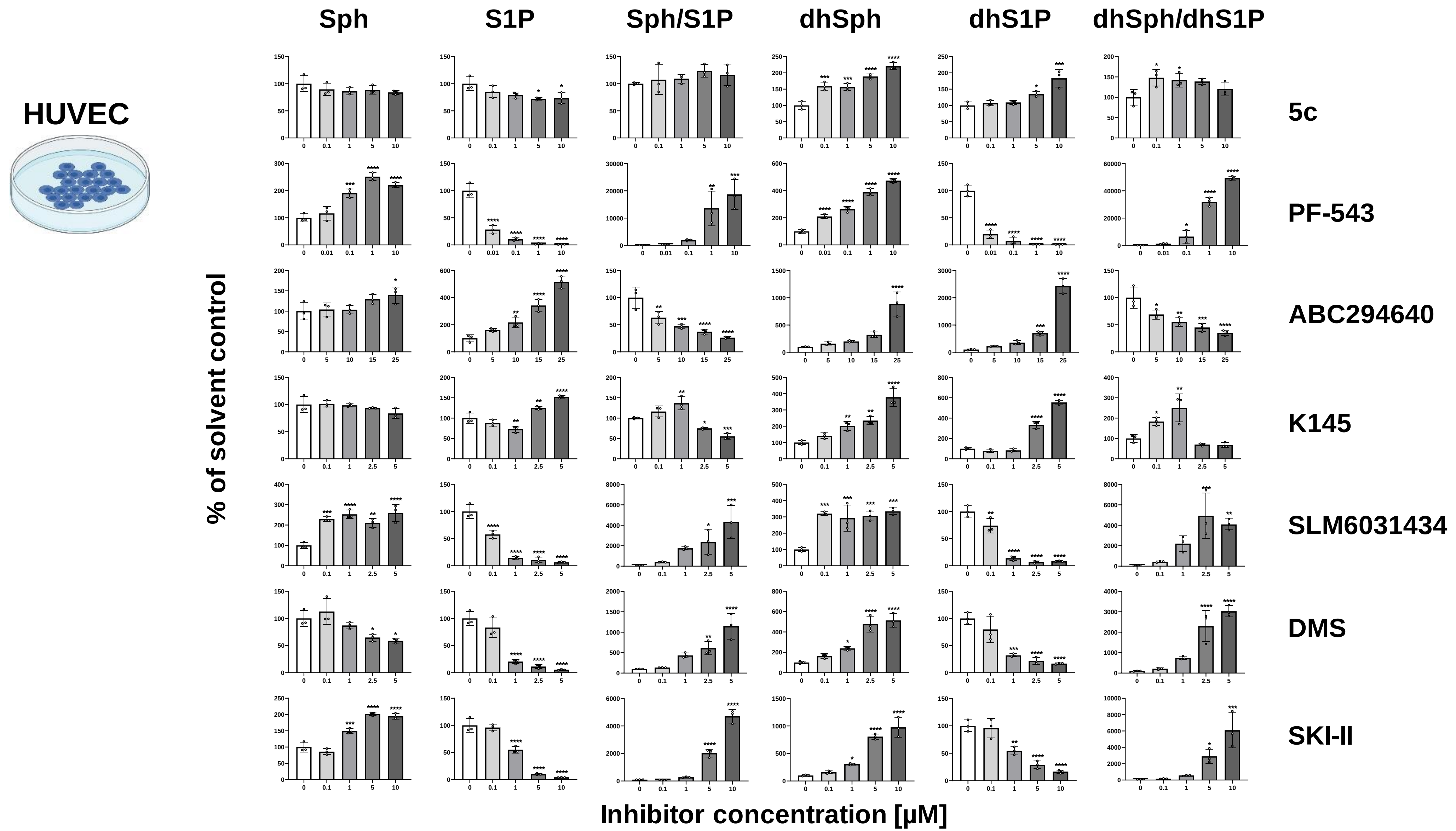

Suppl. Figure 6. Effects of sphingosine kinase inhibitors on the content of sphingosine (Sph), sphingosine 1-phosphate (S1P), dihydrosphingosine (dhSph), dihydrosphingosine 1-phosphate (dhS1P) and selected ratios thereof in HUVEC cells, determined by LC-MS/MS. Cells were treated for 5 h with the indicated concentrations of the inhibitors and quantified amounts were normalized to the solvent control. Shown is the mean ± SD of 3 independent experiments. Statistically significant differences to the solvent control (white bars) were determined using one-way ANOVA with Dunnett's multiple comparison test. *$p<0.05$, **$p<0.01$, ***$p<0.001$, ****$p<0.0001$.

Suppl. Figure 7. Effects of sphingosine kinase inhibitors on the content of dihydroceramide (dhCer) and ceramide (Cer) and on the dhCer/Cer ratio in Chang, HepG2 and HUVEC cells, determined by LC-MS/MS. Cells were treated for 5 h with the indicated concentrations of the inhibitors and quantified amounts were normalized to the solvent control. Shown is the mean ± SD of 3 independent experiments. Statistically significant differences to the solvent control (white bars) were determined using one-way ANOVA with Dunnett's multiple comparison test. *p<0.05, **p<0.01, ***p<0.001, ****p<0.0001.

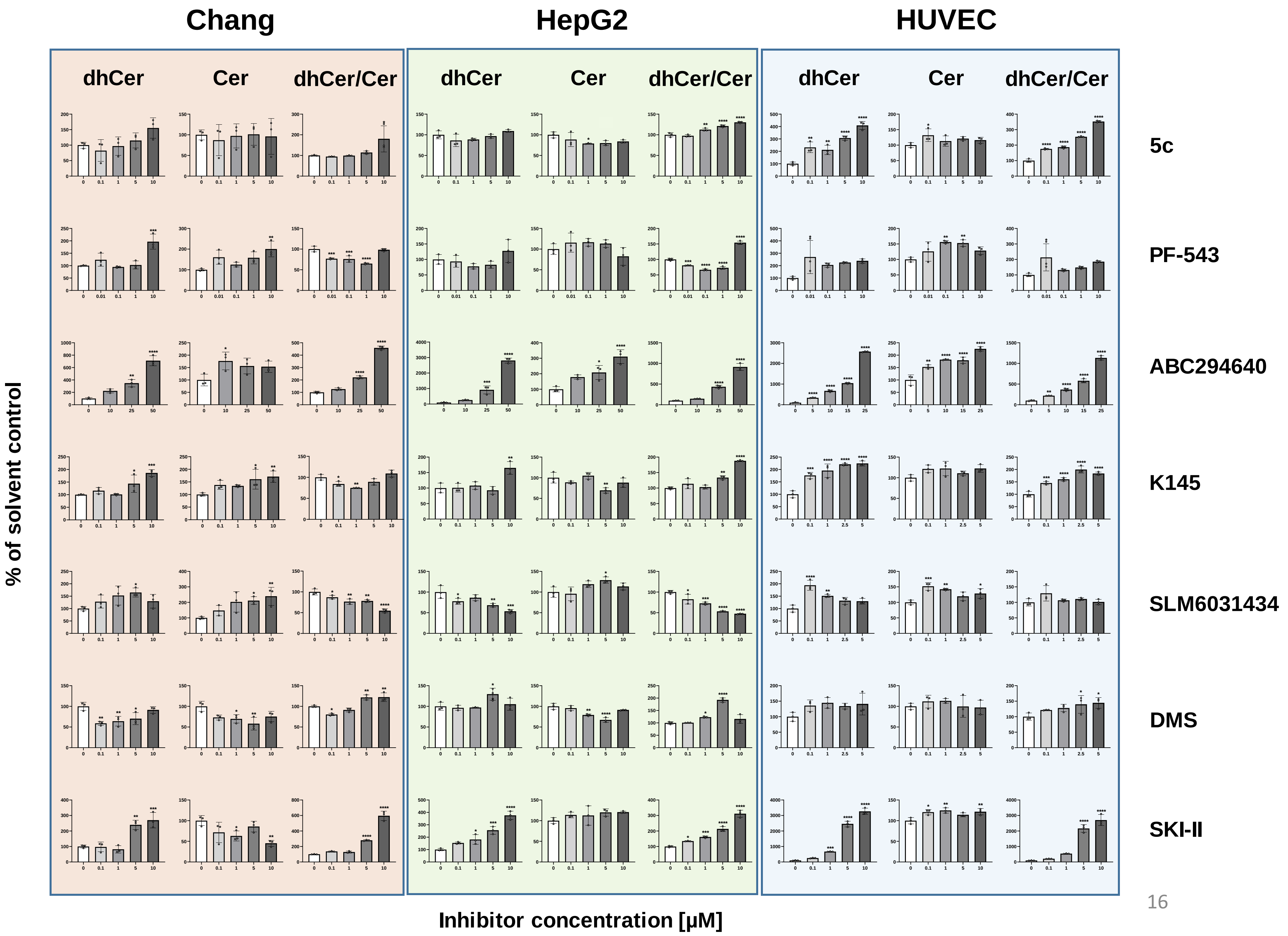

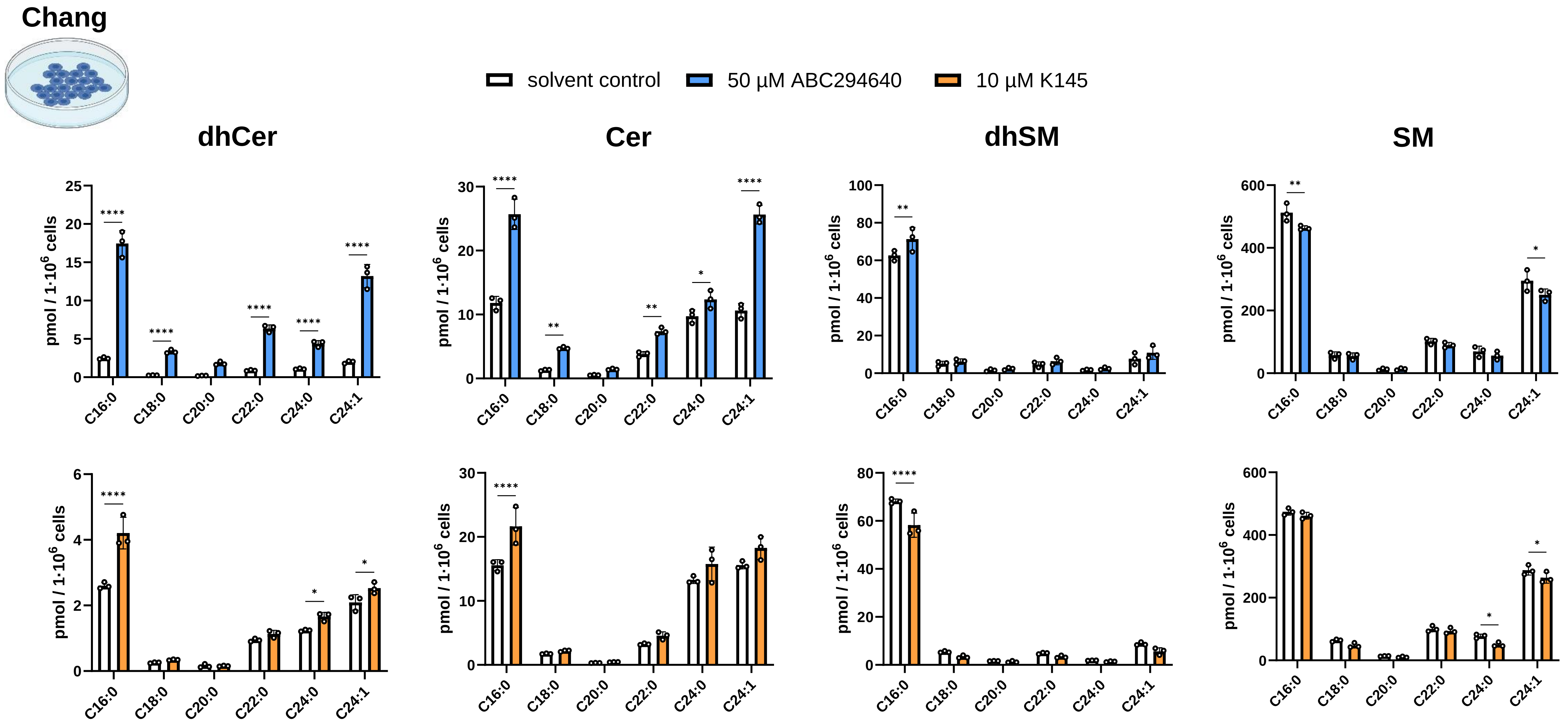

Suppl. Figure 8. Effects of ABC294640 and K145 on the content of dihydroceramide (dhCer), ceramide (Cer), dihydrosphingomyelin (dhSM) and sphingomyelin (SM) subspecies in Chang cells, determined by LC-MS/MS. Cells were treated for 5 h with the indicated concentrations of the inhibitors. The amounts were normalized to cell number. Shown is the mean ± SD of 3 independent experiments. Statistically significant differences to the solvent control (white bars) were determined using two-way ANOVA with Šídák's multiple comparisons test. *p<0.05, **p<0.01, ****p<0.0001.

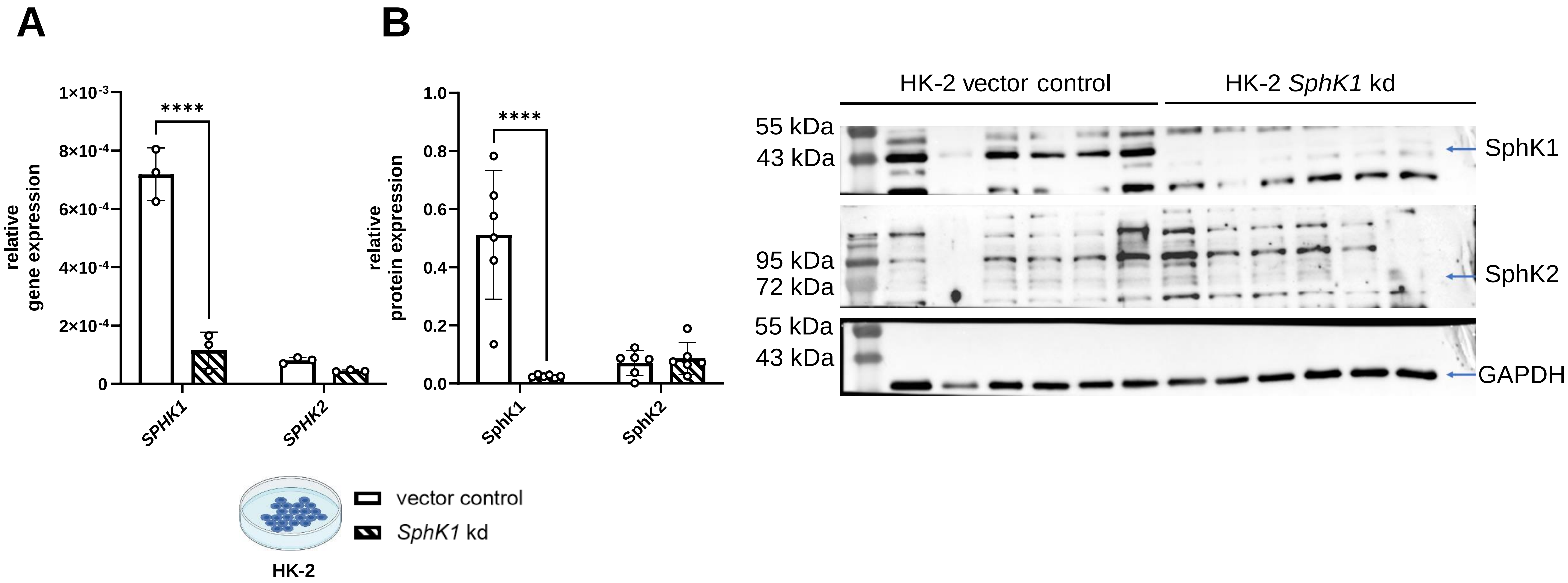

Suppl. Figure 9. A) Relative gene expression of sphingosine kinase 1 (*SPHK1*) and 2 (*SPHK2*) in HK-2 SphK1 knockdown (kd) or vector control cells determined by qPCR. *HMBS* was used as housekeeping gene. Data are mean ± SD of 3 independent experiments. B) SphK1 and SphK2 protein expression in relation to GAPDH in HK-2 SphK1 kd or vector control cells, determined by Western blot. The bar graph shows the quantitative evaluation and on the right are representative blots with labeling of the corresponding protein bands. Data are mean ± SD of 6 independent experiments. Statistically significant differences to the vector control (white bars) were determined using two-way ANOVA with Šídák's test for multiple comparisons. ****p<0.0001.

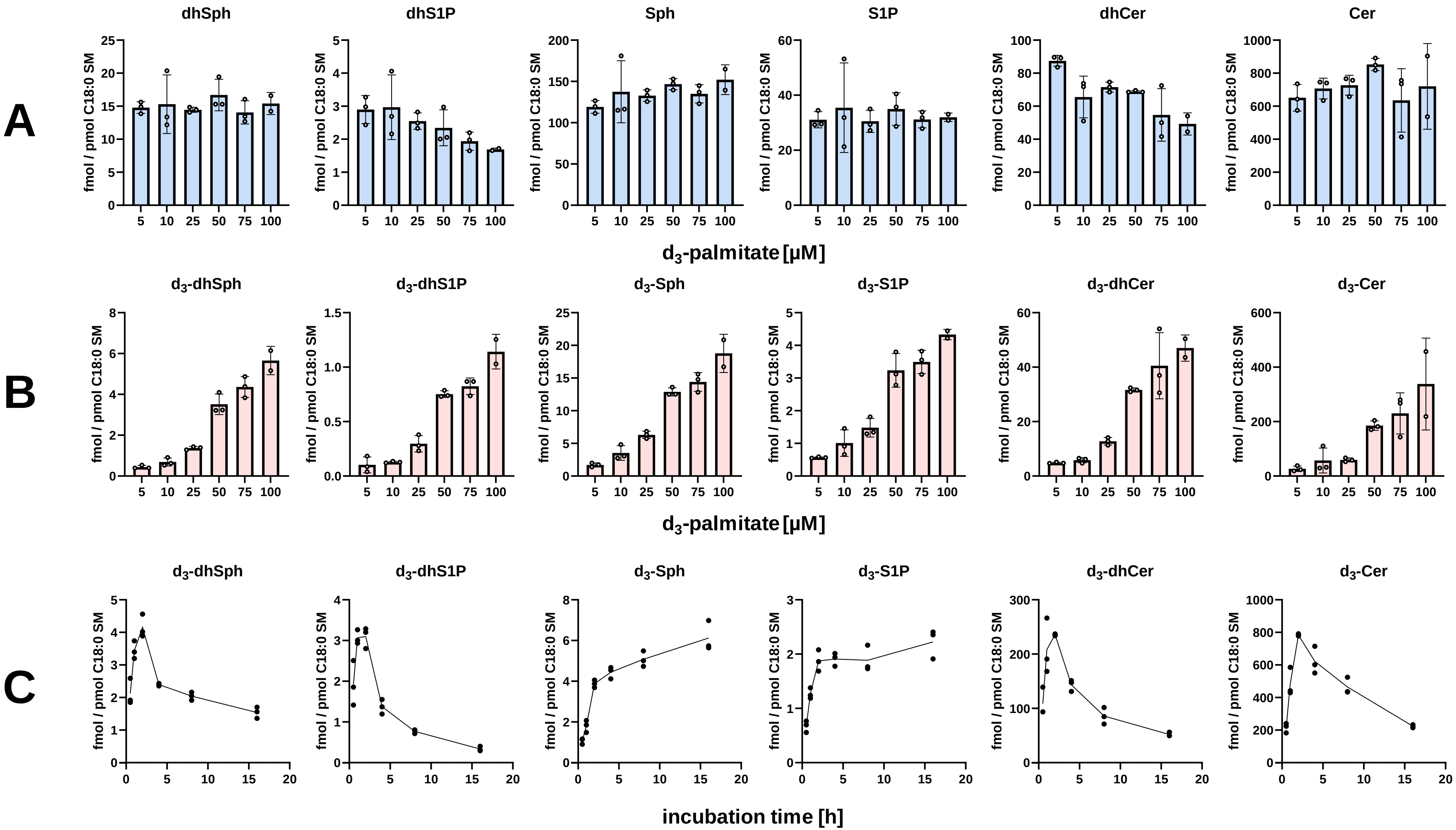

Suppl. Figure 10. Effect of d_3-palmitate incubation on A) intrinsic and B) *de novo* formed sphingolipid levels in Chang cells. Cells were stimulated with the indicated concentrations of d_3-palmitate for 16 h, and sphingolipids (labeled and unlabeled) were analyzed by LC-MS/MS. Determined levels were normalized to C18:0 sphingomyelin (SM). The mean ± SD of 3 independent experiments is shown. C) Time course of *de novo* formed sphingolipids after incubation with 10 µM d_3-palmitate. The values determined by LC-MS/MS were normalized to C18:0 SM. The mean of 3 independent experiments is shown. Dual incorporation of d_3-palmitate also yielded d_6-C16:0 Cer and dhCer species, which are included in the data presented. Cer, ceramide; dhCer, dihydroceramide; dhS1P, dihydrosphingosine 1-phosphate; dhSph, dihydrosphingosine; S1P, sphingosine 1-phosphate; Sph, sphingosine.
